# Mitochondrial Pyruvate Carrier Subunits Are Essential for Pyruvate-Driven Respiration, Infectivity, and Intracellular Replication of Trypanosoma cruzi

**DOI:** 10.1128/mBio.00540-21

**Published:** 2021-04-06

**Authors:** Raquel S. Negreiros, Noelia Lander, Miguel A. Chiurillo, Anibal E. Vercesi, Roberto Docampo

**Affiliations:** a Department of Clinical Pathology, State University of Campinas, Campinas, São Paulo, Brazil; b Center for Tropical and Emerging Global Diseases and Department of Cellular Biology, University of Georgia, Athens, Georgia, USA; c Department of Biological Sciences, University of Cincinnati, Cincinnati, Ohio, USA; Harvard T. H. Chan School of Public Health

**Keywords:** pyruvate carrier, mitochondria, oxygen consumption, *Trypanosoma cruzi*

## Abstract

Pyruvate is the final metabolite of glycolysis and can be converted into acetyl coenzyme A (acetyl-CoA) in mitochondria, where it is used as the substrate for the tricarboxylic acid cycle. Pyruvate availability in mitochondria depends on its active transport through the heterocomplex formed by the mitochondrial pyruvate carriers 1 and 2 (MPC1/MPC2). We report here studies on MPC1/MPC2 of Trypanosoma cruzi, the etiologic agent of Chagas disease. Endogenous tagging of T. cruzi *MPC1* (*TcMPC1*) and *TcMPC2* with 3×*c-Myc* showed that both encoded proteins colocalize with MitoTracker to the mitochondria of epimastigotes. Individual knockout (KO) of *TcMPC1* and *TcMPC2* genes using CRISPR/Cas9 was confirmed by PCR and Southern blot analyses. Digitonin-permeabilized *TcMPC1*-KO and *TcMPC2*-KO epimastigotes showed reduced O_2_ consumption rates when pyruvate, but not succinate, was used as the mitochondrial substrate, while α-ketoglutarate increased their O_2_ consumption rates due to an increase in α-ketoglutarate dehydrogenase activity. Defective mitochondrial pyruvate import resulted in decreased Ca^2+^ uptake. The inhibitors UK5099 and malonate impaired pyruvate-driven oxygen consumption in permeabilized control cells. Inhibition of succinate dehydrogenase by malonate indicated that pyruvate needs to be converted into succinate to increase respiration. *TcMPC1*-KO and *TcMPC2*-KO epimastigotes showed little growth differences in standard or low-glucose culture medium. However, the ability of trypomastigotes to infect tissue culture cells and replicate as intracellular amastigotes was decreased in *TcMPC*-KOs. Overall, T. cruzi MPC1 and MPC2 are essential for cellular respiration in the presence of pyruvate, invasion of host cells, and replication of amastigotes.

## Abstract (precis)

Trypanosoma cruzi is the causative agent of Chagas disease. Pyruvate is the end product of glycolysis, and its transport into the mitochondrion is mediated by the mitochondrial pyruvate carrier (MPC) subunits.

## INTRODUCTION

Pyruvate is the end product of glycolysis and depends on its active transport to reach the mitochondrial matrix. Transport of pyruvate across the outer mitochondrial membrane is by passive diffusion through the large nonselective voltage-dependent anion channel (VDAC) (1, 2). However, transport across the inner mitochondrial membrane is more selective, and the proton-coupled import of the anion pyruvate into the mitochondrial matrix is mediated by a specific mitochondrial pyruvate carrier (MPC) (3–5). Although biochemical studies discovered the presence of an MPC almost 50 years ago, the molecular identification of the MPC has only recently been reported (6, 7).

In the mitochondrial matrix, the pyruvate dehydrogenase (PDH) complex catalyzes the oxidative decarboxylation of pyruvate into acetyl coenzyme A (acetyl-CoA), which is a major substrate for the tricarboxylic acid (TCA) cycle. Therefore, the availability of pyruvate in mitochondria, determined by MPC, links the glycolytic and oxidative phosphorylation pathways and is important for cellular homeostasis.

MPC has been characterized in several organisms (6–10). MPC is generally composed of two main types of small homologous proteins, MPC1 and MPC2. In Saccharomyces cerevisiae, there is a third member of the MPC family, MPC3, which shows high identity with MPC2 in amino acid sequence. Therefore, yeast MPC is constituted by the protomers MPC1 and either MPC2 or MPC3 (6, 7). The MPC1/MPC2 heterocomplex is the active MPC under fermentative conditions, and the MPC1/MPC3 heterocomplex functions under respiratory conditions (11, 12). MPC1 and MPC2 proteins have been reported to migrate as a 150-kDa heterocomplex in blue native polyacrylamide gel electrophoresis (6), but the oligomeric state of the functional MPC has been disputed (13). Yeast MPC has been described as an active heterodimer (MPC1/MPC3 dimer), whereas individual subunits can form homodimers but they are not able to transport pyruvate (13). On the other hand, human MPC2 protomers were able to form high-order MPC2 homocomplexes, which promoted effective pyruvate transport in proteoliposomes independently of MPC1 (14). A posttranslational modification that changes MPC activity has been reported. The MPC1 subunit is deacetylated at lysine residues by Sirt3, and consequently, MPC activity increases (15).

In Trypanosoma brucei, the causative agent of human African trypanosomiasis or sleeping sickness, TbMPC1 and TbMPC2 subunits have been described as essential proteins for mitochondrial pyruvate transport. Both proteins localize to the mitochondrion of procyclic forms (PCF). Knockout of *TbMPC1* and *TbMPC2* genes generated a 2-fold decrease in pyruvate uptake into isolated mitochondria, while the inhibitor UK5099 [2-cyano-3-(1-phenyl-1*H*-indol-3-yl)-2-propenoic acid] reduced mitochondrial pyruvate import in wild-type PCF (9). UK5099 acts on MPC and plasma membrane monocarboxylate transporters (MCTs) (4) but exerts a specific inhibitory effect on MPC in isolated mitochondria or digitonin-permeabilized cells. In bloodstream forms (BSF) of T. brucei, ATP is mainly generated through glycolysis, and most pyruvate is excreted (16) through the pyruvate-proton symporter to maintain the intracellular pH (17). However, PDH activity is present in BSF trypanosomes (18, 19) that also possess an MPC (9). *TbMPC1* knockout (KO) BSF trypanosomes displayed a reduction in growth rate (in Creek’s minimal medium) and in parasitemia and lethality in mice. It was not possible to knockout *TbMPC2* in BSF trypanosomes (9).

Mitochondrial metabolism of pyruvate has been studied in Trypanosoma cruzi (20). PDH is a multienzymatic complex (21) regulated by reversible phosphorylation. Pyruvate dehydrogenase kinase (PDK) phosphorylates PDH (inactive), whereas Ca^2+^-stimulated pyruvate dehydrogenase phosphatase (PDP) dephosphorylates it (active) (22, 23). In T. cruzi, PDP showed mitochondrial localization in epimastigotes. Knockout of T. cruzi PDP (*TcPDP*) resulted in decreased growth rate, metacyclogenesis, infectivity, and mitochondrial oxygen consumption rates (20).

In the present study, we evaluated the importance of T. cruzi MPC (TcMPC) in mitochondrial bioenergetics, growth, differentiation, infectivity, and intracellular replication by individual ablation of *TcMPC1* and *TcMPC2* genes.

## RESULTS

### Sequence analysis.

Two T. cruzi putative proteins, mitochondrial pyruvate carrier 1 (TcMPC1) and mitochondrial pyruvate carrier 2 (TcMPC2), are annotated in TriTrypDB. TcMPC1 (97 amino acids [aa]) and TcMPC2 (137 aa) are small proteins with predicted molecular masses of 11 kDa and 15 kDa, respectively.

TcMPC1 and TcMPC2 proteins have two and three predicted transmembrane domains, respectively (TMpred) (24) (see Fig. S1 in the supplemental material), and mitochondrial targeting sequences with calculated probabilities of mitochondrial import of 0.70 and 0.98, respectively, according to MitoProt II (25). Putative TcMPC proteins are encoded by single-copy genes, *TcMPC1* (gene identifier [ID], TcCLB.511577.144; gene length, 294 bp) and *TcMPC2* (gene ID, TcCLB.508265.44; gene length, 414 bp). There are two *MPC* gene orthologues in T. brucei, *TbMPC1* (gene ID, Tb927.9.3780) and *TbMPC2* (gene ID, Tb927.7.3520), which were previously characterized (9). TcMPC1 and TbMPC1 exhibited 62% and TcMPC2 and TbMPC2 proteins exhibited 75% similarity in amino acid sequence (Fig. S1).

10.1128/mBio.00540-21.1 FIG S1 Amino acid sequence alignments of T. cruzi and T. brucei MPC proteins. (A) Protein alignment of TcMPC1 and TbMPC1. (B) Protein alignment of TcMPC2 and TbMPC2. Pairwise sequence alignments were performed using EMBOSS Needle (https://www.ebi.ac.uk/Tools/psa/emboss_needle/) (59). This online tool uses a space for a mismatch or a gap, “.” for any small positive score, “:” for a similarity which scores more than 1.0, and “|” for an identity where both sequences have the same residue regardless of its score. Red lines represent two predicted transmembrane domains for TcMPC1 (helix 1, amino acids 9 to 27; helix 2, amino acids 37 to 59) and three predicted transmembrane domains for TcMPC2 (helix 1, amino acids 53 to 71; helix 2, amino acids 82 to 98; helix 3, amino acids 103 to 123), according to TMPred (https://embnet.vital-it.ch/software/TMPRED_form.html) (24). Download FIG S1, TIF file, 0.2 MB. Copyright © 2021 Negreiros et al. 2021 Negreiros et al. https://creativecommons.org/licenses/by/4.0/ This content is distributed under the terms of the Creative Commons Attribution 4.0 International license.

### CRISPR/Cas9-mediated endogenous C-terminal tagging of TcMPCs.

**FIG 1:**
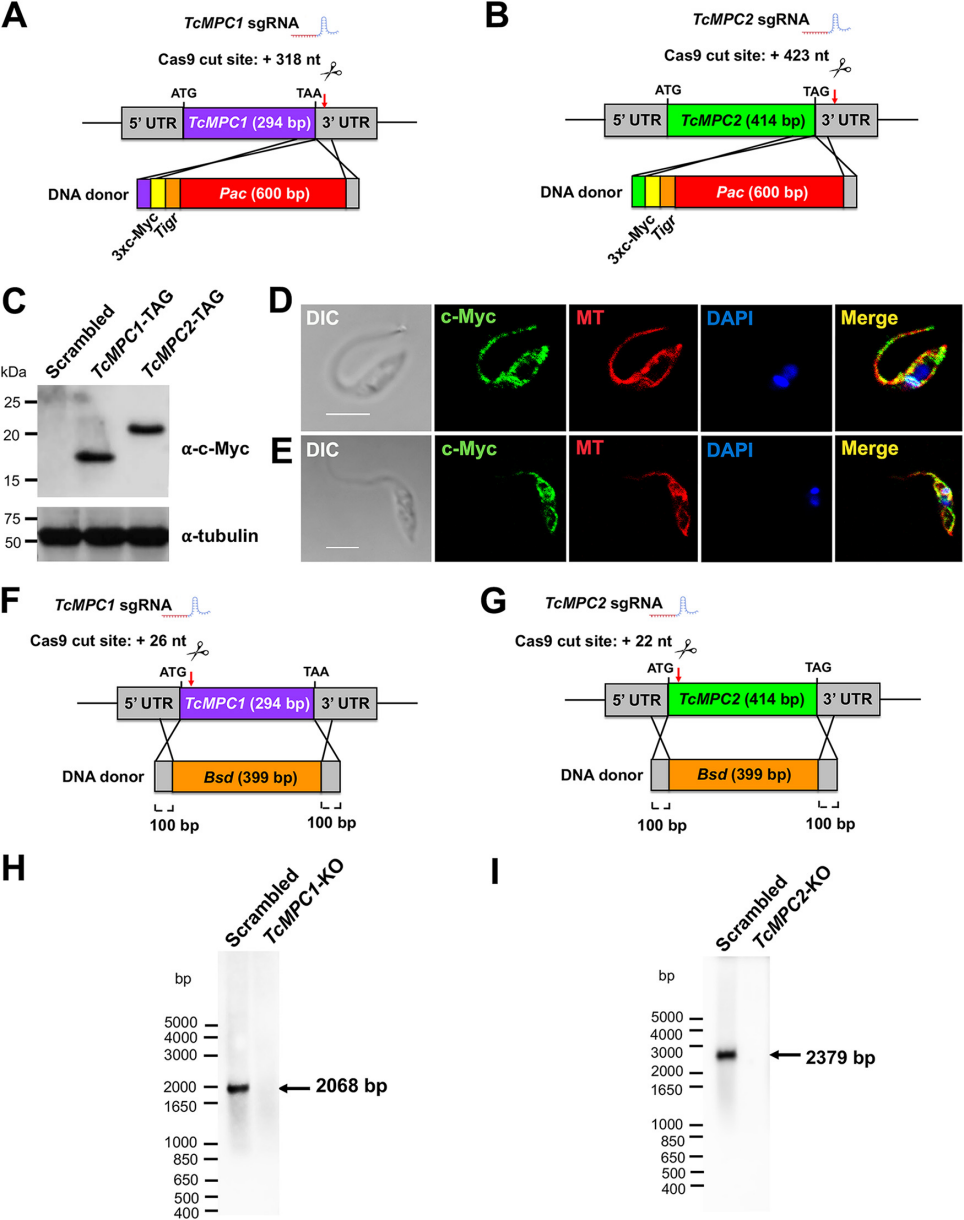
CRISPR/Cas9-induced endogenous tagging and knockout of *TcMPC*s. Schematic representations of individual endogenous tagging of *TcMPC1* (A) and *TcMPC2* (B) genes by homologous recombination. *TcMPC1* and *TcMPC2* sgRNAs direct Cas9 endonuclease to promote double-stranded gDNA break at nucleotide position +318 of the *TcMPC1* 3′ untranslated region (UTR) and nucleotide position 423 of the *TcMPC2* 3′ UTR, respectively. DNA donor cassettes contain two 100-bp homology arms for directed DNA repair, a 3×c-Myc tag (yellow box), and a *Pac* gene (red box, 600 bp), which confers resistance to puromycin. Red arrows indicate Cas9 cut site, and scissors represent Cas9 nuclease. *Tigr*, T. brucei tubulin intergenic region. (C) Western blot analysis of scrambled, *TcMPC1*-TAG, and *TcMPC2*-TAG epimastigotes using monoclonal antibody against c-Myc tag. Predicted protein molecular masses: 17 kDa for TcMPC1-3×c-Myc and 21 kDa for TcMPC2-3×c-Myc. Molecular weight markers are on the left. Tubulin was used as a loading control. Confocal microscope images of epimastigotes expressing TcMPC1-3×c-Myc (D) and TcMPC2-3×c-Myc (E) proteins (green). Both MPC proteins colocalize (merge, yellow) with mitochondrial marker MitoTracker (red). DAPI staining is in blue. Differential interference contrast (DIC) images are shown on the left. Bars, 5 μm. Schematic representations of individual knockout of *TcMPC1* (F) and *TcMPC2* (G) genes by homologous recombination. Double-stranded gDNA break produced by Cas9 is headed by *TcMPC1* and *TcMPC2* sgRNAs at nucleotide position +26 of the *TcMPC1* open reading frame (ORF) and nucleotide position +22 of the *TcMPC2* ORF, respectively. DNA donor cassettes contain two homology arms corresponding to ORF-flanking regions from 5′ and 3′ UTRs, and *Bsd* gene (orange box, 399 bp), which confers resistance to blasticidin. Red arrows indicate Cas9 cut site, and scissors represent Cas9 endonuclease. (H and I) Southern blot analyses for validation of *TcMPC* knockouts. Blots were hybridized with a chemiluminescent probe corresponding to the *TcMPC1* (H) or *TcMPC2* (I) ORF.

To determine their subcellular localization, we endogenously tagged *TcMPC1* (Fig. 1A) and *TcMPC2* (Fig. 1B) using CRISPR/Cas9 gene editing, as described previously (26, 27). A nucleotide sequence encoding a 3×c-Myc tag was inserted at the 3′ ends of *TcMPC1* and *TcMPC2*. The donor sequences contained the tag sequence, the marker for resistance to puromycin (*Pac*, gene encoding puromycin *N*-acetyltransferase, 600 bp), and two 100-bp homology arms.

Tagging of both alleles of *TcMPC1* or *TcMPC2* after 6 weeks of double selection with G418 and puromycin was confirmed by PCR using two specific primer sets for each gene (primers 9 to 16) (see Table S1) designed to discriminate between the intact *TcMPC* locus and the endogenous tagged gene. For *TcMPC1*, PCR analysis using genomic DNA (gDNA) and primers 9 and 10 (Table S1, primer set 1) showed the amplification of a 0.45-kb band in control cell lines (wild-type and transfected with scrambled single guide RNA [sgRNA] [scrambled]) and a 1.7-kb band in the *TcMPC1*-TAG cell line, as expected (see Fig. S2A and B). PCR analysis using primers 11 and 12 (Table S1, primer set 2) also confirmed the endogenous tagging of *TcMPC1* (Fig. S2C and D). For *TcMPC2*, PCR analyses showed expected bands of approximately 0.5-kb in control cell lines and 1.9-kb in the *TcMPC2*-TAG cell line (Fig. S2E and F) using primers 13 and 14 (Table S1, primer set 1), and an expected band of approximately 1.6-kb in *TcMPC2*-TAG cell line using primers 15 and 16 (Table S1, primer set 2) (Fig. S2G and H).

10.1128/mBio.00540-21.2 FIG S2 Schematic representation and PCR validation of endogenous tagging of *TcMPC*s. (A, C, E, and G) Upper schemes represent primers annealing in gDNA from control cell lines (wild type and scrambled). Lower schemes indicate regions for primer annealing in gDNA from the *TcMPC1*-TAG or *TcMPC2*-TAG cell line. Dashed lines indicate PCR products. Two sets of primers were used for each gene. (B, D, F, and H) PCR analyses for validation of *TcMPC* tagging showing expected bands for control cell lines (predicted size, 447 bp) and *TcMPC1*-TAG cell line (predicted size, 1,700 bp) using *TcMPC1* primer set 1 (B), an expected band for *TcMPC1*-TAG (predicted size, 1,396 bp) using *TcMPC1* primer set 2 (D), expected bands for control cell lines (predicted size, 499 bp) and *TcMPC2*-TAG (predicted size, 1,867 bp) using *TcMPC2* primer set 1 (F), and an expected band for *TcMPC2*-TAG (predicted size, 1,589 bp) using *TcMPC2* primer set 2 (H). MW, molecular weight markers. − Ctrl, PCR negative control with ultrapure water. Download FIG S2, TIF file, 0.6 MB. Copyright © 2021 Negreiros et al. 2021 Negreiros et al. https://creativecommons.org/licenses/by/4.0/ This content is distributed under the terms of the Creative Commons Attribution 4.0 International license.

10.1128/mBio.00540-21.8 TABLE S1 List of oligonucleotides used in this study. Download Table S1, DOCX file, 0.02 MB. Copyright © 2021 Negreiros et al. 2021 Negreiros et al. https://creativecommons.org/licenses/by/4.0/ This content is distributed under the terms of the Creative Commons Attribution 4.0 International license.

Western blot analysis confirmed the expression of the proteins TcMPC1-3×c-Myc and TcMPC2-3×c-Myc in their respective cell lines with molecular masses close to their expected values (TcMPC1-3×c-Myc, 17 kDa; TcMPC2-3×c-Myc, 21 kDa) (Fig. 1C). Localization of both proteins to the mitochondrion was confirmed by fluorescence microscopy. Figure 1D and E show colocalization of the green fluorescent signal from the expressed proteins using a monoclonal anti-c-Myc antibody with the mitochondrial marker MitoTracker in red. Thus, our results confirmed that TcMPC1 and TcMPC2 proteins localize to the mitochondria of T. cruzi epimastigotes.

### CRISPR/Cas9-induced knockout of *TcMPCs*.

To investigate the role of the pyruvate carrier, *MPC* knockout cell lines were generated using CRISPR/Cas9 gene editing (28) (Fig. 1F and G). DNA donors contained blasticidin resistance marker (*Bsd*; gene encoding blasticidin S deaminase, 399 bp) and two 100-bp homology arms corresponding to the *TcMPC1* or *TcMPC2* flanking regions. After 6 weeks of selection with G418 and blasticidin, knockouts of *TcMPC1* and *TcMPC2* were validated by PCR and Southern blot analyses.

Three primer sets were designed to carry out PCR analysis using gDNA from doubly resistant parasites as the template. Using primers (primer set 1 [primers 23 and 24], Table S1) designed to discriminate between the intact *TcMPC1* locus and the one replaced by *Bsd*, we amplified bands of approximately 0.75-kb with gDNA from control cell lines and 0.85-kb with gDNA from the *TcMPC1*-KO cell line, indicating the generation of a homogeneous knockout population in which both *TcMPC1* alleles were ablated (Fig. S3A and B). *TcMPC1* knockout was also validated by PCR analysis using different primers (primer set 2 [primers 25 and 26] and primer set 3 [primers 27 and 28], Table S1) (Fig. S3C, D, E, and F). Southern blot analysis using a probe comprising the entire *TcMPC1* (294 bp) also confirmed knockout of both *TcMPC1* alleles, revealing a 2-kb band only for HindIII-digested gDNA from scrambled control cells (Fig. 1H).

10.1128/mBio.00540-21.3 FIG S3 Schematic representation and PCR validation of *TcMPC* knockouts with different primer sets. (A, C, E, G, and I) Upper schemes represent primers annealing in gDNA from control cell lines (wild type and scrambled). Lower schemes indicate regions for primers annealing in gDNA from the *TcMPC1*-KO or *TcMPC2*-KO cell line. Dashed lines indicate PCR products. (B, D, F, H, and J) PCR analyses for validation of *TcMPC* knockouts. Using *TcMPC1* primer set 1, PCR analysis resulted in expected bands for control cell lines (predicted size, 751 bp) and for *TcMPC1*-KO cell line (predicted size, 856 bp) (B). Using *TcMPC1* primer set 2, PCR resulted in an expected band for *TcMPC1*-KO (predicted size, 648 bp) (D). PCR analysis using primer *TcMPC1* set 3 resulted in an expected band for control cell lines (predicted size, 543 bp) (F). Using *TcMPC2* primer set 1, PCR analysis resulted in an expected band for *TcMPC2*-KO (predicted size, 606 bp) (H). Using *TcMPC2* primer set 2, PCR analysis resulted in an expected band for control cell lines (predicted size, 414 bp) (J). − Ctrl, PCR negative control with ultrapure water. Download FIG S3, TIF file, 0.5 MB. Copyright © 2021 Negreiros et al. 2021 Negreiros et al. https://creativecommons.org/licenses/by/4.0/ This content is distributed under the terms of the Creative Commons Attribution 4.0 International license.

A similar strategy was followed to knockout *TcMPC2*. To validate the knockout of *TcMPC2* by PCR, two primer sets were designed. With the first primer set (primer set 1 [primers 29 and 30], Table S1), there was an expected amplification of a 0.6-kb band from gDNA from *TcMPC2*-KO parasites and no amplification for wild-type and scrambled cell lines (Fig. S3G and H). PCR analysis using another primer set (primer set 2 [primers 31 and 32], Table S1) resulted in amplification of a band with expected size of approximately 0.4 kb for control gDNAs (Fig. S3I and J). Using a *TcMPC2* probe (414 bp) for Southern blot analysis, *TcMPC2* knockout was also successful in generating a band of 2.4 kb only for BamHI-digested gDNA from scrambled control cells (Fig. 1I). Further PCR assays were performed to confirm the specific ablation of each *TcMPC* gene (see Fig. S4).

10.1128/mBio.00540-21.4 FIG S4 Confirmation that knockout of one *TcMPC* gene does not affect the other. (A) PCR analysis using *TcMPC1*-KO gDNA (*TcMPC1*^−/−^ *TcMPC2*^+/+^ genotype) and *TcMPC2* primer set 1 showed no amplification, as expected. (B) PCR analysis using *TcMPC1*-KO gDNA (*TcMPC1*^−/−^ *TcMPC2*^+/+^ genotype) and *TcMPC2* primer set 2 showed an expected band for all control and *TcMPC1*-KO cell lines (predicted size, 414 bp). (C) Using *TcMPC2*-KO gDNA (*TcMPC1*^+/+^ *TcMPC2*^−/−^ genotype) and *TcMPC1* primer set 1, there was amplification of an expected band for all control and *TcMPC2*-KO cell lines (predicted size, 751 bp). (D) PCR analysis using *TcMPC2*-KO gDNA (*TcMPC1*^+/+^ *TcMPC2*^−/−^ genotype) and *TcMPC1* primer set 2 showed no amplification, as expected. (E) PCR analysis using *TcMPC2*-KO gDNA (*TcMPC1*^+/+^ *TcMPC2*^−/−^ genotype) and *TcMPC1* primer set 3 showed amplification of an expected band for all control and *TcMPC2*-KO cell lines (predicted size, 543 bp). − Ctrl, PCR negative control with ultrapure water. Download FIG S4, TIF file, 0.5 MB. Copyright © 2021 Negreiros et al. 2021 Negreiros et al. https://creativecommons.org/licenses/by/4.0/ This content is distributed under the terms of the Creative Commons Attribution 4.0 International license.

### Effect of *TcMPC*-KOs on epimastigote growth.

**FIG 2:**
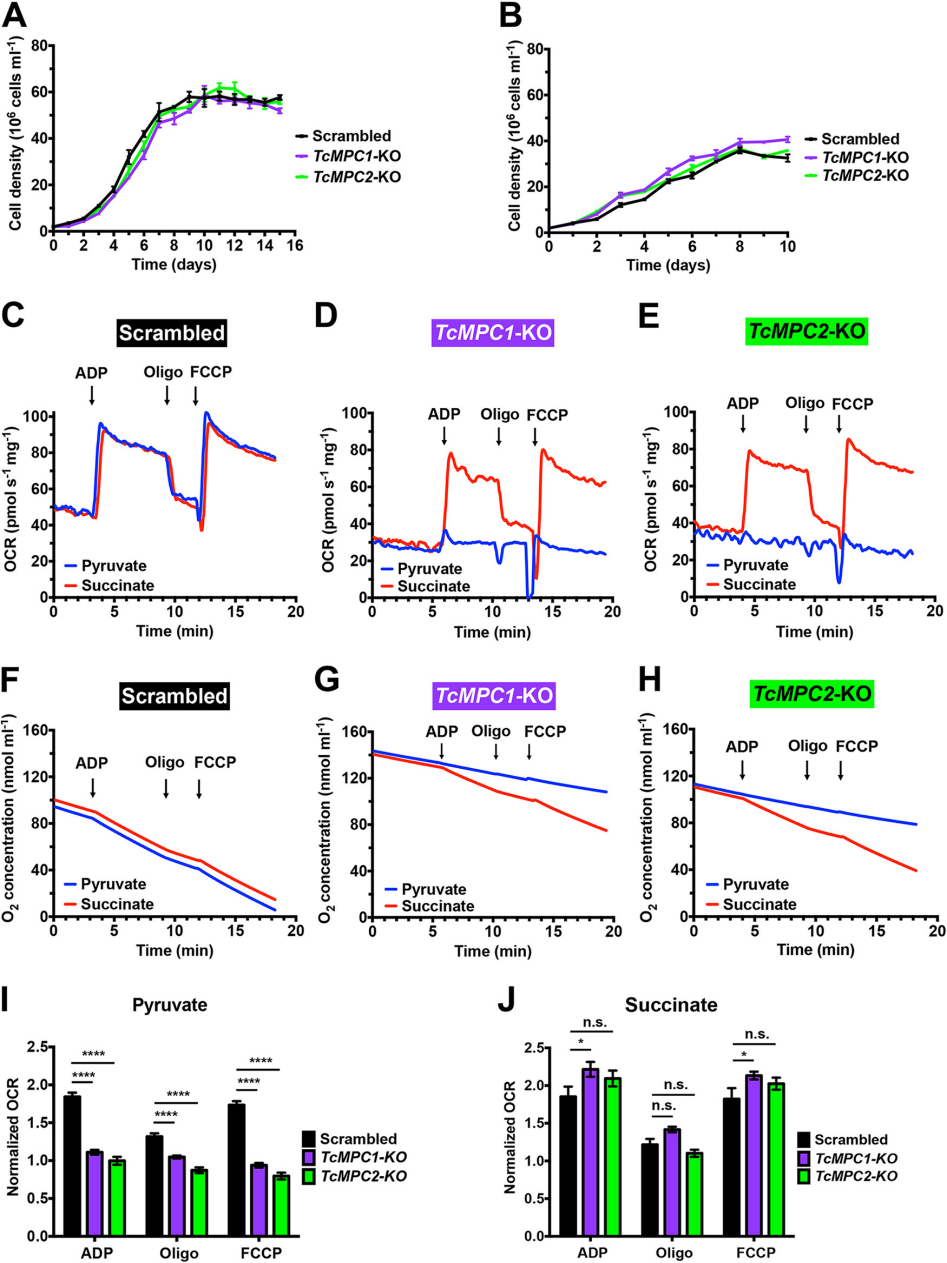
Phenotypic changes of *TcMPC*-KO parasites. Growth curves of *TcMPC1*-KO (purple) and *TcMPC2*-KO (green) epimastigotes compared to scrambled cells (black) in standard LIT medium over 15 days (A) and in low-glucose LIT medium over 10 days (B). Representative OCR (C to E) and oxygen concentration (F to H) tracings of digitonin-permeabilized scrambled (C and F), *TcMPC1*-KO (D and G), and *TcMPC2*-KO (E and H) epimastigotes when pyruvate (blue lines) or succinate (red lines) was used as the mitochondrial substrate. Additions of 100 μM ADP (respiration stimulated by oxidative phosphorylation [OXPHOS]), 1 μg ml^−1^ oligomycin (Oligo, minimal respiratory rate), and 1 μM FCCP (maximal respiratory capacity) are indicated by arrows. Normalized OCRs upon addition of ADP, oligomycin, and FCCP when pyruvate (I) and succinate (J) were used as the substrate. Normalized OCR of 1 means that OCR upon an addition is equal to the basal OCR (prior to ADP addition). Values are means ± SEMs (*n *= 6). *, *P* < 0.05; ****, *P* < 0.0001; n.s., no significant differences. Two-way ANOVA test with multiple comparisons.

To assess the role of MPC proteins in epimastigote growth, *TcMPC1*-KO and *TcMPC2*-KO epimastigotes were grown in liver infusion tryptose (LIT) medium in the absence or presence of additional glucose and compared to control parasites (transfected with scrambled sgRNA). There was no significant reduction in the growth rates of both knockout epimastigotes in either standard (Fig. 2A) or low-glucose (Fig. 2B) LIT medium.

### Effect of *TcMPC*-KOs on stimulation of mitochondrial oxygen consumption.

To demonstrate that pyruvate could not be transported to the mitochondria in the knockout parasites, we measured oxygen consumption by mitochondria *in situ* of digitonin-permeabilized epimastigotes in the presence of pyruvate or succinate as the respiratory substrate. Oxygen consumption rates (OCRs) were evaluated under ADP-stimulated (state 3, respiration stimulated by oxidative phosphorylation [OXPHOS]), oligomycin-inhibited (state 4, minimal respiratory rate), and carbonyl cyanide 4-(trifluoromethoxy) phenylhydrazone (FCCP)-stimulated (state 3u [uncoupled], maximal respiratory capacity) conditions and compared to those in parasites transfected with the scrambled sgRNA (control cells).

Control cells showed similar OCR (Fig. 2C) and oxygen uptake (Fig. 2F) tracings in the presence of either pyruvate (blue lines) or succinate (red lines) as the substrate. In contrast, in the presence of pyruvate, both *TcMPC*-KO mutants showed only basal OCRs upon additions of ADP, oligomycin, and FCCP (Fig. 2D, E, and I) and diminished oxygen uptake (Fig. 2G and H). However, *TcMPC1*-KO and *TcMPC2*-KO mutants showed comparable OCRs to those of the control cells upon additions of ADP, oligomycin, and FCCP when succinate was used (Fig. 2D, E, and J). These results suggest that both TcMPC1 and TcMPC2 are important for mitochondrial pyruvate import.

To rule out any influence of the potential presence of endogenous substrates in the preparations, we also measured oxygen consumption in the absence of exogenous substrates. Under these conditions, there was no OCR stimulation by ADP, and the basal OCRs were maintained in control and knockout cells (see Fig. S5). Addition of the mitochondrial complex III inhibitor antimycin A that results in an interruption of the electron flux from cytochrome *b* to cytochrome *c*_1_ decreased OCR to almost zero (Fig. S5).

10.1128/mBio.00540-21.5 FIG S5 Endogenous substrates do not stimulate OCRs of digitonin-permeabilized *TcMPC*-KO epimastigotes. Representative OCR tracings of digitonin-permeabilized scrambled (A), *TcMPC1*-KO (B), and *TcMPC2*-KO (C) epimastigotes with no exogenous substrates. Additions of ADP (respiration stimulated by oxidative phosphorylation [OXPHOS]), oligomycin (Oligo, minimal respiratory rate), FCCP (maximal respiratory capacity), and antimycin A are indicated by arrows. Download FIG S5, TIF file, 0.1 MB. Copyright © 2021 Negreiros et al. 2021 Negreiros et al. https://creativecommons.org/licenses/by/4.0/ This content is distributed under the terms of the Creative Commons Attribution 4.0 International license.

**FIG 3:**
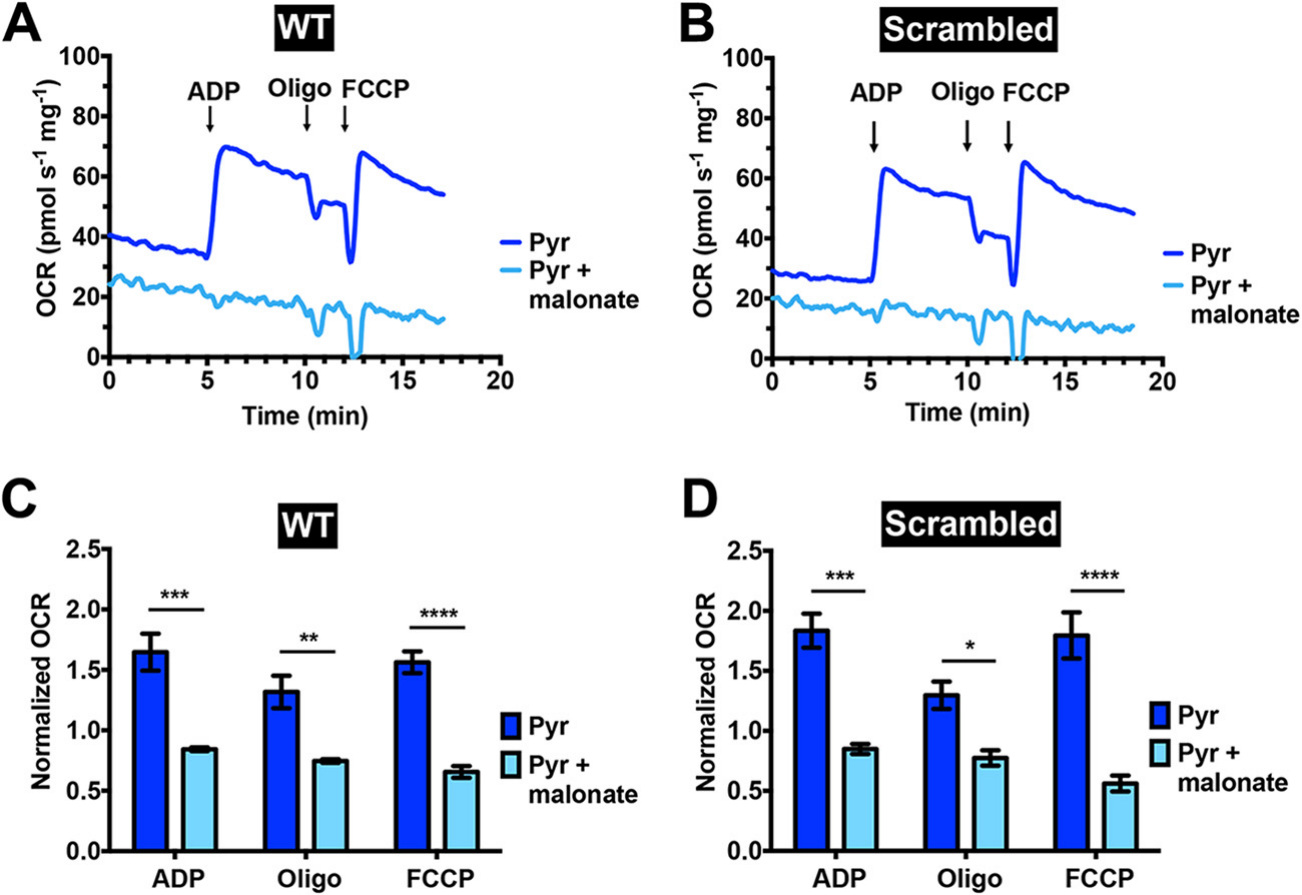
Complex II inhibitor malonate impaired oxygen consumption in digitonin-permeabilized control epimastigotes in the presence of pyruvate. Representative OCR tracings of digitonin-permeabilized wild-type (WT) (A) and scrambled (B) epimastigotes in the absence (dark blue lines) or presence (light blue lines) of malonate (2 mM) when pyruvate was used as a respiratory substrate. Normalized OCRs upon addition of 100 μM ADP, 1 μg ml^−1^ oligomycin, and 1 μM FCCP for wild-type (C) and scrambled (D) epimastigotes. Normalized OCR of 1 means that OCR upon an addition is equal to the basal OCR (prior to ADP addition). Values are means ± SEMs (*n *= 3). *, *P* < 0.05; **, *P* < 0.01; ***, *P* < 0.001; ****, *P* < 0.0001. Two-way ANOVA test with multiple comparisons.

Pyruvate is commonly used as an electron donor for complex I of the respiratory chain through the formation of NADH catalyzed by PDH. However, complex I appears to be functionally limited in T. cruzi (29), although pyruvate was able to stimulate oxygen consumption in permeabilized cells as well as succinate (Fig. 2C). To investigate whether this stimulation was due to the previous conversion of pyruvate into succinate, we measured oxygen consumption driven by pyruvate in the absence or presence of malonate. Malonate is a competitive inhibitor of succinate dehydrogenase, which is a component of complex II localized at the matrix face of the inner mitochondrial membrane. Succinate dehydrogenase catalyzes the conversion of succinate into fumarate, and the resultant reduced flavin adenine dinucleotide (FADH_2_) feeds electrons into complex II. OCR analysis of digitonin-permeabilized control cells (wild-type and cells transfected with scrambled sgRNA) showed a complete inhibition of pyruvate-driven oxygen consumption when malonate was added to the reaction medium (light blue lines and bars, Fig. 3), indicating that pyruvate needs to be converted into succinate in order to donate electrons to the respiratory chain.

### Inhibition of TcMPC by UK5099 decreases pyruvate-driven respiration.

**FIG 4:**
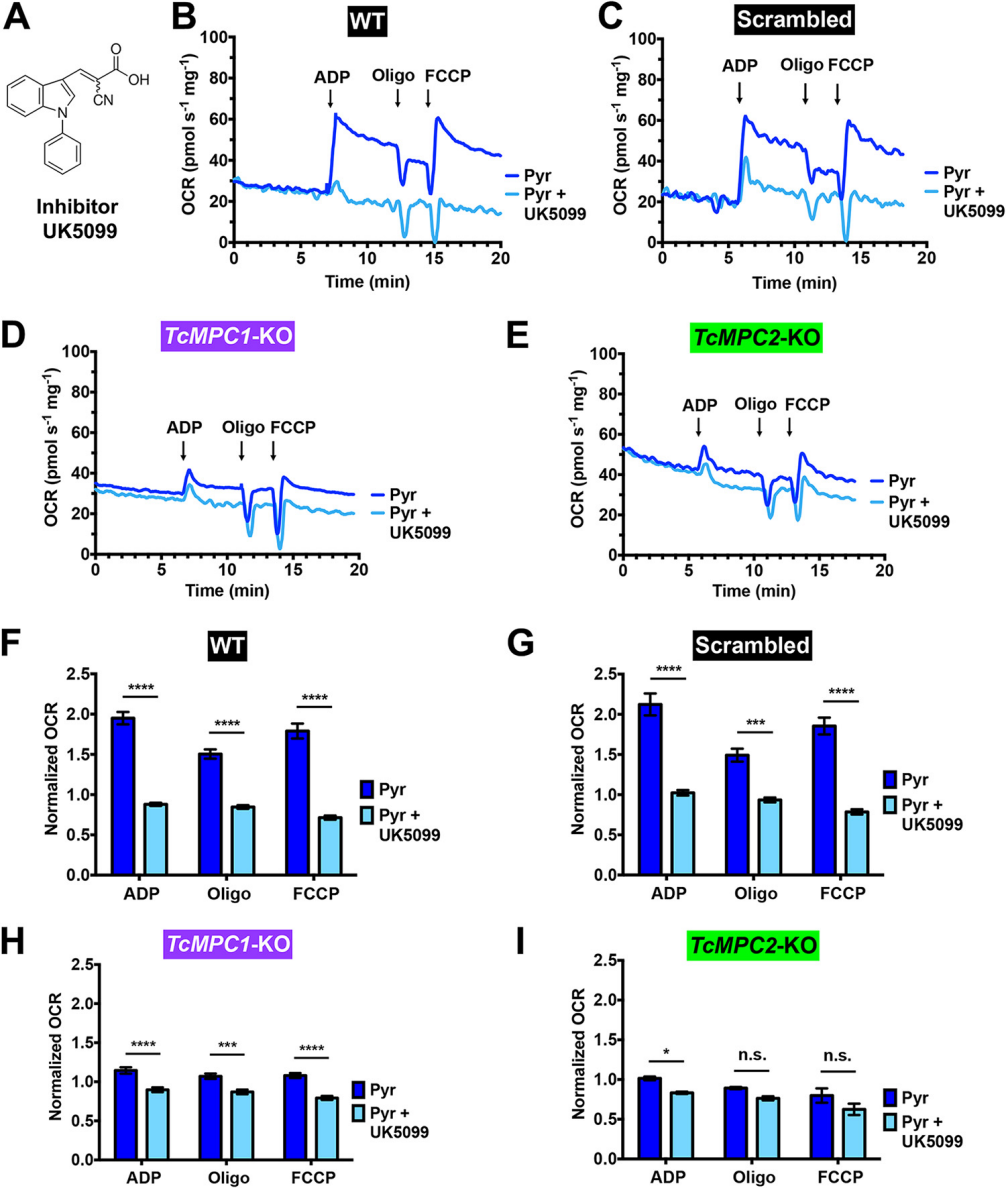
Inhibitor UK5099 impaired oxygen consumption in digitonin-permeabilized control epimastigotes in the presence of pyruvate. (A) Molecular structure of UK5099. Representative OCR tracings of digitonin-permeabilized wild-type (WT) (B), scrambled (C), *TcMPC1*-KO (D), and *TcMPC2*-KO (E) epimastigotes in the absence (dark blue lines) or presence (light blue lines) of UK5099 (50 μM) when pyruvate was used as a respiratory substrate. Normalized OCRs upon addition of 100 μM ADP, 1 μg ml^−1^ oligomycin, and 1 μM FCCP for wild-type (F), scrambled (G), *TcMPC1*-KO (H), and *TcMPC2*-KO (I) epimastigotes. Normalized OCR of 1 means that OCR upon an addition is equal to the basal OCR (prior to ADP addition). Values are means ± SEMs (*n *= 5). *, *P* < 0.05; ***, *P* < 0.001; ****, *P* < 0.0001; n.s., no significant differences. Two-way ANOVA test with multiple comparisons.

We tested whether pharmacological inhibition of TcMPC by an inhibitor of MCTs and MPC, UK5099 (Fig. 4A) (4), resulted in similar changes in pyruvate-driven respiration to those observed in the knockout epimastigotes. We tested different concentrations of the inhibitor (10, 25, and 50 μM) on permeabilized cells and found the best inhibition with 50 μM UK5099 (see Fig. S6). Addition of this concentration of UK5099 significantly decreased the OCRs of wild-type cells and cells transfected with scrambled sgRNA in the presence of ADP, oligomycin, and FCCP (light blue lines and bars in Fig. 4B, C, F, and G), while it had little effect on the *TcMPC*-KO mutants (Fig. 4D, E, H, and I).

10.1128/mBio.00540-21.6 FIG S6 Inhibition of pyruvate-driven respiration by UK5099 in digitonin-permeabilized control epimastigotes. Representative OCR tracings of digitonin-permeabilized wild-type (WT) (A) and scrambled (B) epimastigotes when pyruvate was used as a respiratory substrate, in the absence (blue lines) or presence of different concentrations (10, 25, and 50 μM) of UK5099. Additions of ADP (respiration stimulated by oxidative phosphorylation), oligomycin (Oligo, minimal respiratory rate), and FCCP (maximal respiratory capacity) are indicated by arrows. Normalized OCRs upon addition of ADP, oligomycin, and FCCP for wild-type (C) and scrambled (D) epimastigotes. Normalized OCR of 1 means that OCR upon an addition is equal to the basal OCR (prior to ADP addition). Download FIG S6, TIF file, 0.4 MB. Copyright © 2021 Negreiros et al. 2021 Negreiros et al. https://creativecommons.org/licenses/by/4.0/ This content is distributed under the terms of the Creative Commons Attribution 4.0 International license.

### Effects of *TcMPC*-KOs on α-ketoglutarate consumption.

**FIG 5:**
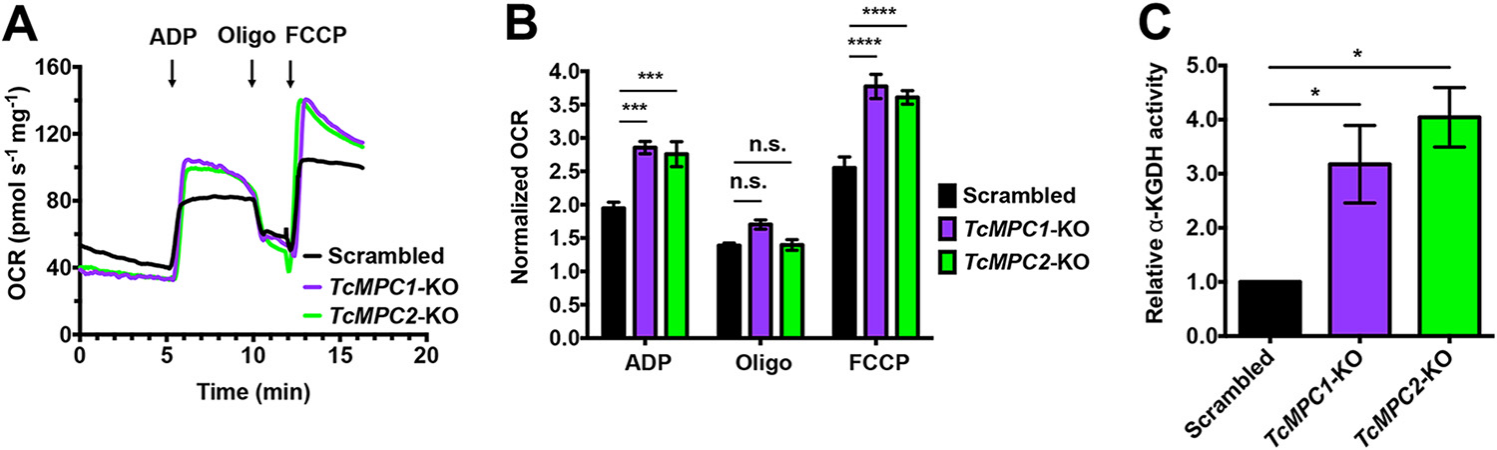
α-Ketoglutarate increases oxygen consumption in digitonin-permeabilized *TcMPC*-KO epimastigotes. (A) Representative OCR tracings of digitonin-permeabilized scrambled, *TcMPC1*-KO, and *TcMPC2*-KO epimastigotes when α-ketoglutarate was used as a respiratory substrate. (B) Normalized OCRs upon addition of 100 μM ADP, 1 μg ml^−1^ oligomycin, and 1 μM FCCP. Normalized OCR of 1 means that OCR upon an addition is equal to the basal OCR (prior to ADP addition). Values are means ± SEMs (*n *= 3). ***, *P* < 0.001; ****, *P* < 0.0001; n.s., no significant differences. Two-way ANOVA test with multiple comparisons. (C) α-Ketoglutarate dehydrogenase (α-KGDH) activity in lysates from scrambled, *TcMPC1*-KO, and *TcMPC2*-KO epimastigotes. Values are means ± SEMs (*n *= 3). *, *P* < 0.05. One-way ANOVA test with multiple comparisons.

It has been reported that when the mammalian MPC is deficient, increased glutaminolysis can compensate for the deficit in pyruvate-derived carbon to fuel the TCA cycle (30–32). Glutamine is converted into glutamate by a glutaminase, and then glutamate is converted into α-ketoglutarate, a TCA cycle metabolite. However, a glutaminase gene orthologue to the human enzyme is apparently missing from the T. cruzi genome (33), and other sources of glutamate might be available. We therefore tested whether α-ketoglutarate synthesis and utilization were altered in the *MPC*-KO mutant cells. We first measured changes in cellular respiration in digitonin-permeabilized control, *TcMPC1*-KO, and *TcMPC2*-KO cells upon addition of ADP, oligomycin, and FCCP in the presence of α-ketoglutarate as the substrate (Fig. 5A and B). Both *TcMPC*-KO mutant epimastigotes showed increased respiration stimulated by OXPHOS and maximal respiratory capacity compared to those of control cells (Fig. 5A and B). In agreement with these results, α-ketoglutarate dehydrogenase (α-KGDH) activity in lysates from *TcMPC*-KO mutants was higher than that of control cells (Fig. 5C).

### PDH phosphorylation and Ca^2+^ uptake in *TcMPC*-KO parasites.

**FIG 6:**
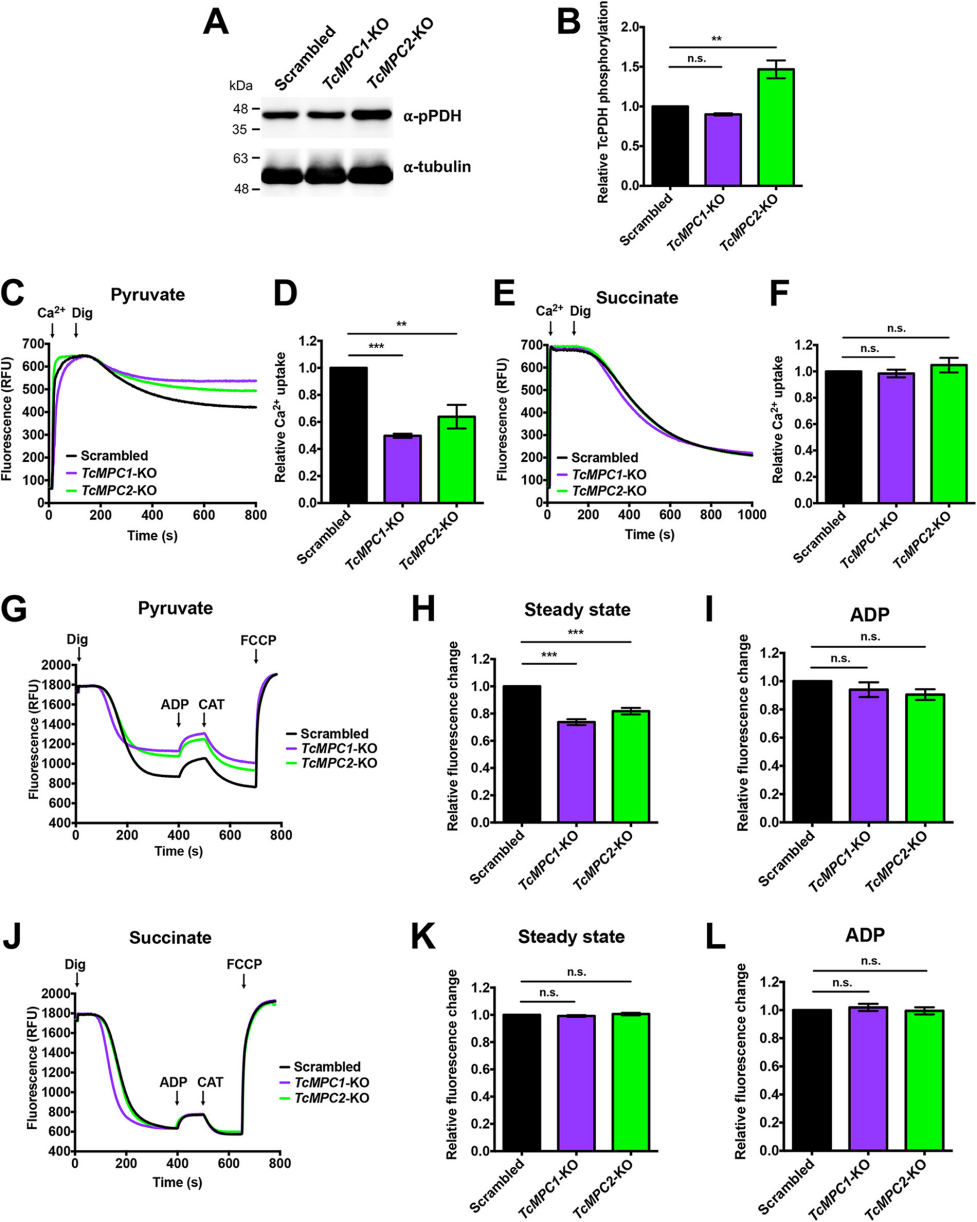
Mitochondrial Ca^2+^ uptake and membrane potential of control and *MPC* knockout epimastigotes. (A) Representative Western blot for TcPDH E1α subunit phosphorylation (commercial anti-PDH E1α subunit antibody [phosphopeptide Ser-293]) in lysates from scrambled, *TcMPC1*-KO, and *TcMPC2*-KO epimastigotes. Molecular weight markers are on the left. Tubulin was used as a loading control. (B) Densitometry analysis of independent Western blots for TcPDH E1α subunit phosphorylation (as in panel A). Values are means ± SEMs (*n *= 3). **, *P* < 0.01; n.s., no significant differences. One-way ANOVA test with multiple comparisons. (C and E) Ca^2+^ uptake by digitonin-permeabilized scrambled, *TcMPC1*-KO, and *TcMPC2*-KO epimastigotes in relative fluorescence units (RFU). The reaction was started after adding 50 μM digitonin in the presence of 20 μM free Ca^2+^, 0.5 μM calcium green-5N probe, and 5 mM pyruvate (C) or succinate (E). (D and F) Quantification of data in panels C and E. Relative Ca^2+^ uptake at 600 s compared with control (scrambled) epimastigotes. Values are means ± SEMs (*n* = 3). **, *P* < 0.01; ***, *P* < 0.001; n.s., no significant differences. One-way ANOVA test with multiple comparisons. (G and J) Changes in mitochondrial membrane potential (Δψ_m_) of digitonin-permeabilized epimastigotes as detected by changes in safranine O fluorescence in scrambled, *TcMPC1*-KO, and *TcMPC2*-KO cells. Cells (5 × 10^7^) were added to the reaction buffer containing 0.2% BSA, 50 μM EGTA, 5 μM safranine O, and 5 mM pyruvate (G) or succinate (J). The reaction was started with 50 μM digitonin, and 250 μM ADP, 1.5 μM CAT, and 4 μM FCCP were added as indicated. CAT was added to inhibit the ADP/ATP translocase and prevent recycling of ADP produced by the ATPase activity of the ATP synthase. Relative changes in safranine O fluorescence upon addition of digitonin (steady state, 350 s), in the presence of pyruvate (H) or succinate (K). Values are mean ± SEMs (*n* = 3). ***, *P* < 0.001; n.s., no significant differences. One-way ANOVA test with multiple comparisons. Relative changes in safranine O fluorescence upon addition of ADP, in the presence of pyruvate (I) or succinate (L). Values are means ± SEMs (*n* = 3)., n.s., no significant differences. One-way ANOVA test with multiple comparisons.

We reported previously that T. cruzi PDH E1α subunit of the PDH complex (PDC) is stimulated by dephosphorylation by a Ca^2+^-stimulated PDP (20). It has also been reported that decreased expression of MPC2 in Alzheimer’s disease-related models led to decreased Ca^2+^ and pyruvate uptake into mitochondria and reduced Ca^2+^-mediated stimulation of the TCA cycle, although the reason for the decrease in Ca^2+^ import was unknown (34). We therefore tested the phosphorylation state of the PDH E1α subunit in lysates from knockout mutants by Western blotting using a commercial antibody against the PDH E1α subunit (phosphopeptide Ser-293) of human cells that recognizes the phosphorylated site 1 (Ser-284) of the T. cruzi enzyme (34). Our results indicate that T. cruzi PDH phosphorylation is higher in *TcMPC2*-KO epimastigotes than in control and *TcMPC1*-KO parasites (Fig. 6A and B).

Using pyruvate as mitochondrial substrate, 2- and 1.5-fold decreases in mitochondrial Ca^2+^ uptake were detected in digitonin-permeabilized *TcMPC1*-KO and *TcMPC2*-KO epimastigotes, respectively, compared to that in the control cells (Fig. 6C and D). However, when succinate was used as the substrate, Ca^2+^ uptake was not affected in either *TcMPC1*-KO or *TcMPC2*-KO mutants (Fig. 6E and F). Therefore, defective mitochondrial import of pyruvate resulted in decreased Ca^2+^ uptake.

We then used safranine O to evaluate mitochondrial membrane potential (Δψ_m_) in digitonin-permeabilized epimastigotes in the presence of pyruvate or succinate as the mitochondrial substrate. A decrease in fluorescence intensity after permeabilization with digitonin indicates stacking of the dye to the energized inner mitochondrial membrane. Fluorescence intensity in the steady state was decreased in both knockout epimastigotes in the presence of pyruvate (Fig. 6G and H), but not succinate (Fig. 6J and K), evidencing an alteration in the mitochondrial membrane potential of knockout parasites when pyruvate was used as the substrate. However, phosphorylation of ADP was not affected in either case (Fig. 6I and L).

### Effect of *TcMPC*-KOs on the infective stages.

**FIG 7:**
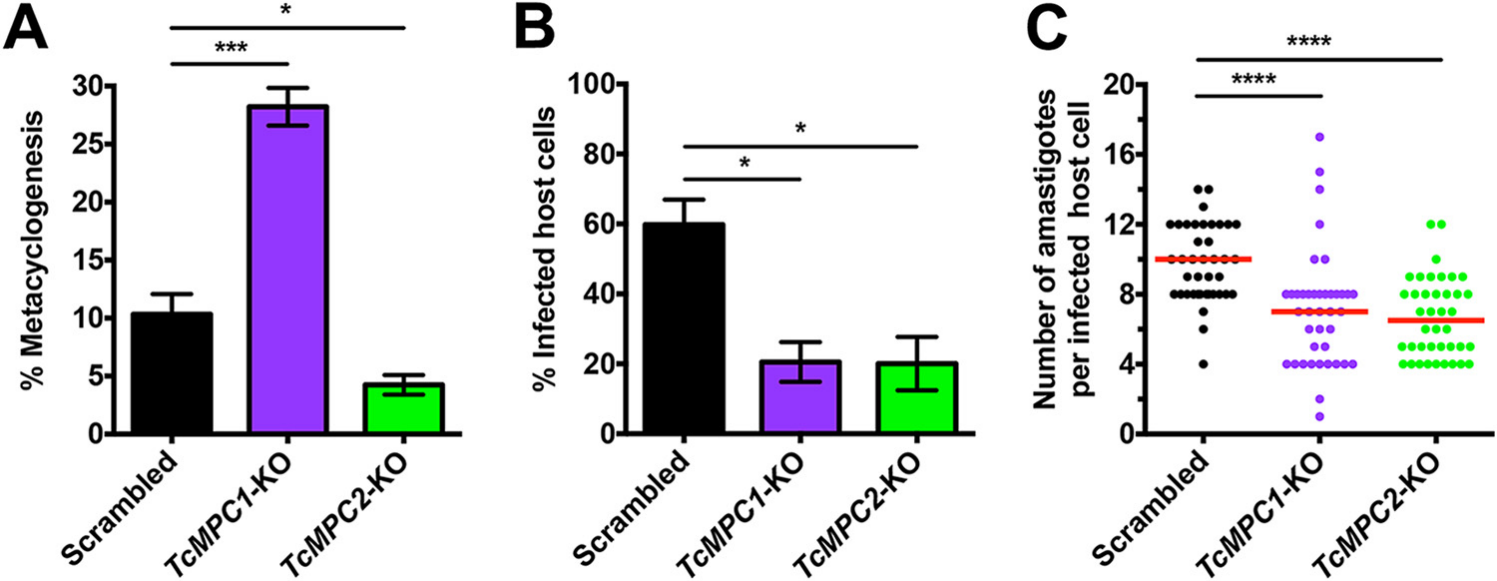
Effect of *TcMPC*-KOs on the infective stages. (A) Percentages of metacyclogenesis of *TcMPC1*-KO and *TcMPC2*-KO parasites after incubation in TAU 3AAG medium compared to that for scrambled cells. Quantification of metacyclic trypomastigotes differentiated from epimastigotes by the kinetoplast position in relation to the nucleus (DAPI staining). Values are means ± SEMs (*n *= 3). *, *P* < 0.05; ***, *P* < 0.001. One-way ANOVA test with multiple comparisons. (B) Ability of *TcMPC1*-KO and *TcMPC2*-KO trypomastigotes to infect tissue cultured mammalian cells is 3-fold reduced compared to that of scrambled control trypomastigotes. Values are means ± SEMs (*n *= 3). *, *P* < 0.05. One-way ANOVA test with multiple comparisons. (C) Quantification of intracellular replication of *TcMPC1*-KO and *TcMPC2*-KO amastigotes 48 h postinfection. Red lines correspond to the medians from one representative experiment (*n *= 40 per cell line). ****, *P* < 0.0001. Kruskal-Wallis test with Dunn's multiple comparisons. Three experiments were performed and the results were reproducible.

The ability of *TcMPC*-KO epimastigotes to differentiate *in vitro* into metacyclic trypomastigotes was also analyzed. Interestingly, the percentage of metacyclogenesis was approximately 3-fold higher in *TcMPC1*-KO parasites and 2-fold lower in *TcMPC2*-KO parasites than in parasites transfected with a scrambled sgRNA (Fig. 7A), suggesting other potential functions for these carriers. The infectivity of both *TcMPC1*-KO and *TcMPC2*-KO trypomastigotes was approximately 3-fold lower than that of scrambled control trypomastigotes (Fig. 7B). Moreover, the replication of intracellular amastigotes was also significantly impaired for both *TcMPC*-KO cell lines (Fig. 7C). Therefore, TcMPC subunits are important for differentiation, host cell invasion, and intracellular replication.

### Overexpression of TcMPC proteins.

We also tested whether individual overexpression of the TcMPC subunits with a 3× hemagglutinin (HA) tag produced significant phenotypic changes. Both expressed proteins had molecular masses close to their expected values (TcMPC1-3×HA, 17 kDa; TcMPC2-3×HA, 21 kDa), as indicated by Western blotting (see Fig. S7A). *TcMPC*-OE epimastigotes did not grow at a higher rate compared to the control parasites (transfected with pTREX-n/3×HA empty vector [EV]) (Fig. S7B). Immunofluorescence analysis showed a mitochondrial localization for both proteins (Fig. S7C and S7D). Slight changes were detected in the OCRs in oxygen consumption stimulated by OXPHOS, minimal respiratory rate, and maximal respiratory capacity using pyruvate as the mitochondrial substrate (Fig. S7E and F).

10.1128/mBio.00540-21.7 FIG S7 Overexpression of TcMPC proteins slightly affects cell bioenergetics. (A) Western blot analysis of control (transfected with pTREX-n/3×HA empty vector [EV]), *TcMPC1*-OE, and *TcMPC2*-OE epimastigotes using monoclonal antibody against the HA tag. Predicted protein molecular masses: 17 kDa for TcMPC1-3×HA and 21 kDa for TcMPC2-3×HA. Molecular weight markers are on the left. Tubulin was used as a loading control. (B) Growth curves of *TcMPC1*-OE (light red line) and *TcMPC2*-OE (orange line) epimastigotes compared to that of control empty vector parasites (dark red line) in LIT medium over 10 days. Confocal microscope images of epimastigotes expressing TcMPC1-3×HA (C) and TcMPC2-3×HA (D) proteins (green). Both MPC proteins colocalize (merge, yellow) with mitochondrial marker MitoTracker (red). DAPI staining is in blue. Differential interference contrast (DIC) images are shown on the left. Bars, 5 μm. (E) Representative OCR tracings of digitonin-permeabilized control empty vector, *TcMPC1*-OE, and *TcMPC2*-OE epimastigotes when pyruvate was used as mitochondrial substrate. (F) Normalized OCRs upon addition of 100 μM ADP, 1 μg ml^−1^ oligomycin, and 1 μM FCCP. Normalized OCR of 1 means that OCR upon an addition is equal to the basal OCR (prior to ADP addition). Values are means ± SEMs (*n *= 3). * P < 0.05; n.s., no significant differences. Two-way ANOVA test with multiple comparisons. Download FIG S7, TIF file, 0.7 MB. Copyright © 2021 Negreiros et al. 2021 Negreiros et al. https://creativecommons.org/licenses/by/4.0/ This content is distributed under the terms of the Creative Commons Attribution 4.0 International license.

## DISCUSSION

Our studies indicate that both MPC1 and MPC2 localize to the mitochondrion of T. cruzi where they have an essential role in pyruvate stimulation of respiration, with previous conversion of pyruvate into succinate. Individual knockout of each subunit results in ablation of this activity, suggesting that the function of one subunit cannot be compensated by the presence of the other and therefore both proteins must be present. Epimastigotes deficient in any of the two subunits have no growth deficiencies in either standard or low-glucose LIT medium, but trypomastigotes are severely impeded in their ability to invade host cells and replicate intracellularly as amastigotes.

**FIG 8:**
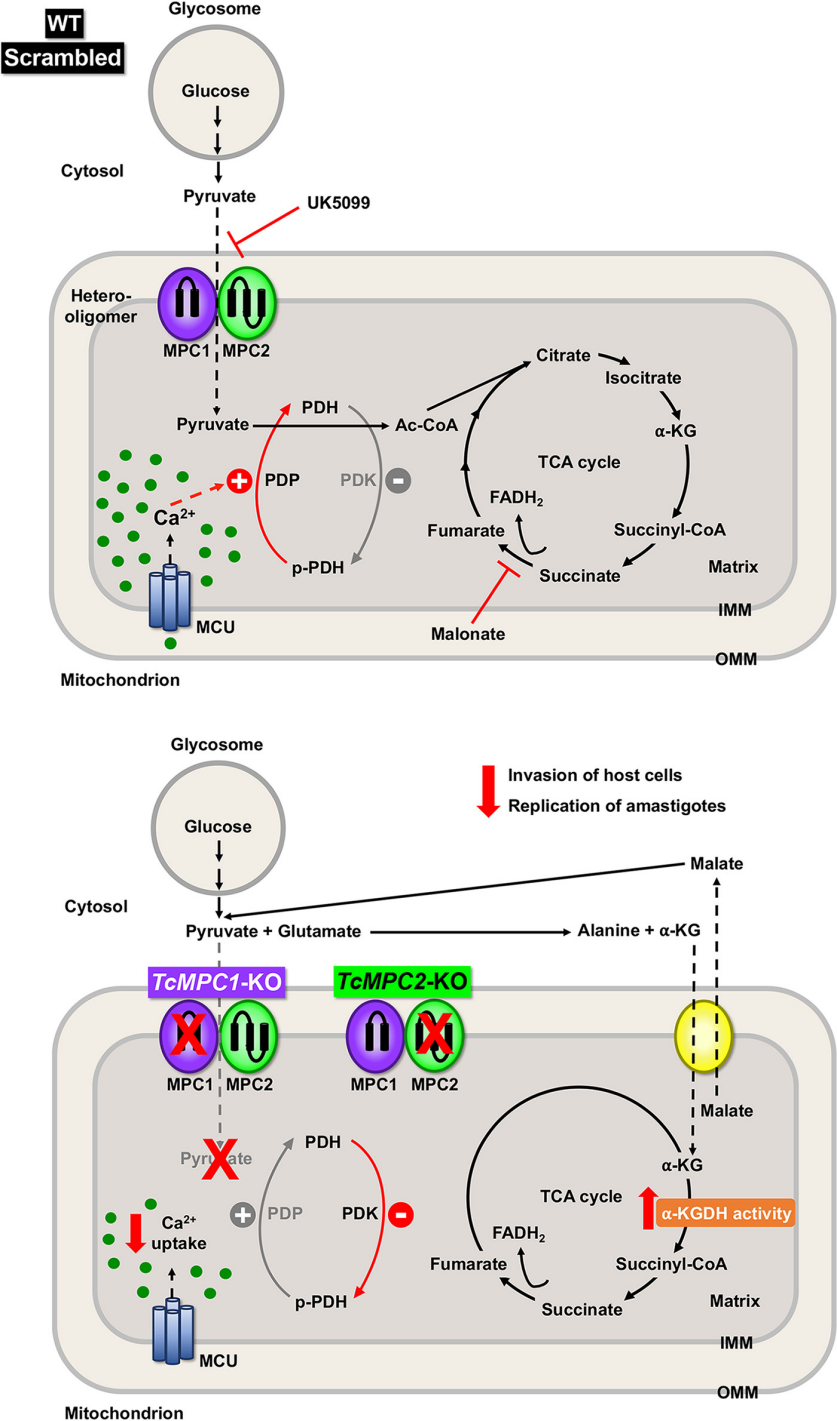
Schematic representation of pyruvate metabolism in control and *TcMPC*-KO parasites. Pyruvate is actively transported into the mitochondrion through MPC. TcMPC is a heterooligomer composed of two small integral membrane proteins, TcMPC1 and TcMPC2, which have two and three predicted transmembrane domains, respectively. In the mitochondrion of control cells (top), pyruvate import is inhibited by UK5099. Ca^2+^ uptake through mitochondrial calcium uniporter (MCU) induces dephosphorylation of PDH by Ca^2+^-stimulated PDP, thus activating PDH. Pyruvate is converted into acetyl-CoA (Ac-CoA) by PDH in the mitochondrial matrix, and succinate is generated from several reactions of the TCA cycle. Succinate is converted into fumarate that supports electron flux through complex II of the respiratory chain via FADH_2_, as demonstrated by our findings of a decrease in oxygen consumption by the succinate dehydrogenase inhibitor malonate. Individual knockout of *TcMPC1* and *TcMPC2* (bottom) impairs pyruvate transport across the inner mitochondrial membrane (IMM), causing accumulation of pyruvate in the cytosol. Pyruvate and glutamate are converted into alanine and α-ketoglutarate (α-KG) by alanine aminotransferase. α-KG is transported to the mitochondrion through the α-ketoglutarate carrier (yellow) that exchanges α-KG for malate. Pyruvate can then be regenerated from cytosolic malate by the malic enzyme. The increase in oxygen consumption with α-ketoglutarate is in agreement with increased α-KGDH activity in *TcMPC*-KO epimastigotes. Ca^2+^ uptake is diminished in the mitochondria of knockout cells, which decreases phosphatase activity of PDP, and consequently, this leads to a higher PDH phosphorylation by PDK (in at least *TcMPC2*-KO cells). Knockout of *TcMPC*s in infective stages resulted in decreased ability to infect host cells and replicate as intracellular amastigotes. Solid arrows indicate enzymatic reactions, and dashed black arrows represent transport of metabolites through mitochondrial carriers. OMM, outer mitochondrial membrane.

In T. cruzi epimastigotes, pyruvate can enter the mitochondrion to be converted into acetyl-CoA and feed the tricarboxylic acid cycle or can be converted into alanine in the cytosol by the reaction catalyzed by alanine aminotransferase or in the glycosome by the action of an l-alanine dehydrogenase, with reoxidation of NADH needed to balance its reduction by glyceraldehyde 3-phosphate dehydrogenase (35). Although T. cruzi epimastigotes have a high rate of glucose consumption, they are characterized by the production and excretion of partially reduced products of glucose metabolism, such as succinate, alanine, and acetate, instead of oxidizing glucose completely to CO_2_ and water (35–37). This metabolism is known as “aerobic fermentation of glucose” and could be responsible for the lack of growth defects in the MPC1/MPC2 knockout mutants: instead of being used for the TCA cycle, pyruvate could contribute to the maintenance of the redox balance in the glycosomes or could be excreted as alanine, while the increased α-ketoglutarate consumption, revealed by the increased activity of α-ketoglutarate dehydrogenase and the increase in respiration by α-ketoglutarate, could replace the pyruvate needed for the TCA cycle. It is possible that cytosolic accumulation of pyruvate leads to its reaction with glutamate catalyzed by alanine aminotransferase, resulting in the formation of α-ketoglutarate and alanine. Glutamate and alanine are the most abundant amino acids present in epimastigotes (38). α-Ketoglutarate could then enter the mitochondrion through the α-ketoglutarate carrier that exchanges α-ketoglutarate for malate and that has been characterized in T. brucei (39, 40) (T. cruzi gene orthologue, TriTrypDB gene ID, TcCLB.509805.190). Cytosolic malate can then be converted into pyruvate catalyzed by the malic enzyme. The malic enzyme is stimulated by aspartate (41) and, as a result, amino acid production would stimulate a reaction for the efficient utilization of amino acids for energy. This combination of reactions, shown in Fig. 8, would produce α-ketoglutarate that enters the TCA cycle and alanine that could be excreted.

A different situation would occur with the trypomastigote and amastigote stages. Attachment to host cells and their invasion by trypomastigotes are active processes that require energy, and it has been shown that glycolysis (2-deoxyglucose) or respiratory chain (antimycin A) inhibitors prevent these activities (42), which perhaps could not be compensated for by increased α-ketoglutarate consumption. Lack of MPC1 or MPC2 appears to affect less the intracellular replication of amastigotes (approximately 1.5-fold reduced) than the mammalian host cell invasion by trypomastigotes (approximately 3-fold reduced). This is in agreement with our results with TcPDP (20). The predominance of fatty acid oxidation and amino acid oxidation (43, 44) over glycolysis has been proposed for these intracellular stages, although it has recently been reported that they are also able to use glucose (45). Amastigotes can also use glutamate from the host cells (33).

An important difference in the functionality of the respiratory chain between the mitochondrion of T. cruzi and the mitochondria of mammalian cells is the limited function of complex I in the parasite (29). Our results indicate that pyruvate needs to be converted into succinate in order to provide electrons to the respiratory chain and are in agreement with the limited role of complex I. It has been suggested that reducing equivalents enter the respiratory chain as succinate through the action of an NADH-dependent fumarate reductase (46, 47).

We found that *TcMPC1*-KO and *TcMPC2*-KO epimastigotes have decreased Ca^2+^ uptake into the mitochondrion when pyruvate, but not when succinate, was used as the substrate. A decrease in MPC2 expression in Alzheimer's disease models is associated with less Ca^2+^ uptake (34), and studies in hepatocytes and embryonic fibroblasts have shown the Ca^2+^ import into mitochondria is also decreased following inhibition of MPC activity (48). In the latter case, the effect is mediated by increased expression of mitochondrial calcium uptake 1 (MICU1), which together with MICU2, is the gatekeeper of the mitochondrial calcium uniporter (MCU). However, in T. cruzi epimastigotes, overexpression of either MICU1 or MICU2 does not affect Ca^2+^ import into the mitochondrion (49), and the reason for this lower Ca^2+^ uptake could be the lower mitochondrial membrane potential when pyruvate is used as the substrate. Decreased mitochondrial Ca^2+^ uptake in *TcMPC2*-KO cells could explain the higher phosphorylation of the TcPDH E1α subunit due to decreased phosphatase activity of the Ca^2+^-stimulated PDP. The effect of the KOs on mitochondrial respiration is then expected to be due to a diminished flux from pyruvate to the TCA cycle that is affected by both the lower mitochondrial uptake and the presence of inactive PDH.

Interestingly, the higher phosphorylation of the E1α subunit was only detected in *TcMPC2*-KO but not in *TcMPC1*-KO cells. Since these experiments reveal the phosphorylation state of PDH *in vivo*, they suggest that epimastigotes are using more pyruvate than succinate under physiological conditions, and when *TcMPC2* is knocked out, there is higher phosphorylation of the TcPDH E1α subunit because pyruvate is not transported to the mitochondrial matrix. The lack of increased E1α subunit phosphorylation in *TcMPC1*-KO cells could be due to the selection of clones that depend more on succinate than on pyruvate as the mitochondrial substrate, and there is no difference in Ca^2+^ uptake when succinate is the substrate.

Our results are compatible with the formation of functional heterooligomers of TcMPC1 and TcMPC2, in contrast with the postulated formation of active homocomplexes of human MPC2 (14). When yeast MPC1/MPC2 were originally identified, it was reported that blue native polyacrylamide gel electrophoresis showed that both proteins migrated as part of an ∼150-kDa complex, and it was concluded that MPC1 and MPC2 form a multimeric complex, with MPC2 as the major structural subunit (6). However, recent work has found no evidence of a multimeric complex and characterized them as a heterodimer (13).

In summary, the mitochondrial pyruvate carrier of T. cruzi is essential for pyruvate-driven respiration, trypomastigote invasion of host cells, and intracellular amastigote replication.

## MATERIALS AND METHODS

### Chemicals and reagents.

Heat-inactivated fetal bovine serum (FBS) and penicillin-streptomycin were from Vitrocell (Campinas, Brazil). G418 was from KSE Scientific (Durham, NC). Puromycin, blasticidin S HCl, Platinum *Taq* DNA polymerase high fidelity, subcloning efficiency DH5α competent cells, and MitoTracker Deep Red FM were from Invitrogen (Eugene, OR). Antarctic phosphatase, BamHI, XbaI, XhoI, and HindIII restriction enzymes were from New England BioLabs (Ipswich, MA). T4 DNA ligase, GoTaq G2 Flexi DNA polymerase, Wizard Plus SV miniprep purification system, Wizard SV gel, and PCR clean-up system were from Promega (Madison, WI). Four-millimeter electroporation cuvettes, Zeta-Probe GT membrane, Precision Plus protein dual color standards, and nitrocellulose membranes were from Bio-Rad (Hercules, CA). North2South chemiluminescent hybridization and detection kit, North2South biotin random prime labeling kit, bicinchoninic acid (BCA) protein assay kit, SuperSignal West Pico chemiluminescent substrate, mouse anti-HA monoclonal antibody, and calcium green-5N were from Thermo Fisher Scientific (Waltham, MA). Mouse anti-c-Myc monoclonal antibody (9E10) was from Santa Cruz Biotechnology (Dallas, TX). Horseradish peroxidase-conjugated anti-mouse IgG antibody and Alexa Fluor 488-conjugated goat anti-mouse were from Life Technologies (Grand Island, NY). Fluoromount-G mounting medium was from Southern Biotech (Birmingham, AL). UK5099 was from Tocris Bioscience (Abingdon, United Kingdom). The pMOTag23M vector (50) was a gift from Thomas Seebeck (University of Bern, Bern, Switzerland). DNA oligonucleotides were purchased from Exxtend Biotecnologia Ltd. (Campinas, Brazil). Phenylmethylsulfonyl fluoride (PMSF), *N*-*p*-tosyl-l-phenylalanine chloromethyl ketone (TPCK), *trans*-epoxysuccinyl-l-leucylamido-(4-guanidino)butane (E64), protease inhibitor cocktail for use with mammalian cell and tissue extracts (catalog number P8340), mouse anti-tubulin monoclonal antibody, digitonin, ADP, oligomycin A, carboxyatractyloside (CAT), carbonyl cyanide 4-(trifluoromethoxy) phenylhydrazone (FCCP), antimycin A, safranine O, malonate, sodium pyruvate, succinate, α-ketoglutarate, Benzonase nuclease, and all other reagents of analytical grade were from Sigma-Aldrich (St. Louis, MO).

### Cell cultures.

T. cruzi epimastigotes (Y strain) were grown at 28°C in liver infusion tryptose (LIT) medium (51) supplemented with 10% heat-inactivated fetal bovine serum (FBS), penicillin (100 U ml^−1^), and streptomycin (100 μg ml^−1^). LIT medium contained 2 g liter^−1^ glucose. To prepare a low-glucose LIT medium, glucose was not added. The glucose in the supplemented low-glucose LIT medium (approximate glucose concentration of 0.07 g liter^−1^) was from the FBS. Cell density was verified by counting parasites using Muse cell analyzer (Millipore, Burlington, MA). Parasites from the control cell lines transfected with scrambled sgRNA (scrambled) or pTREX-n-3×HA empty vector (EV), and overexpressing (OE) cell lines *TcMPC1*-OE and *TcMPC2*-OE were grown in the presence of 250 μg ml^−1^ G418. *TcMPC1*-TAG and *TcMPC2*-TAG cell lines were maintained with 250 μg ml^−1^ G418 and 5 μg ml^−1^ puromycin. *TcMPC1*-KO and *TcMPC2*-KO cell lines were grown with 250 μg ml^−1^ G418 and 10 μg ml^−1^ blasticidin. Tissue culture-derived trypomastigotes were collected from the culture medium of infected Vero cells, using a modification of the method of Schmatz and Murray (1982) as previously described (52). Vero cells were grown in RPMI medium supplemented with 10% FBS and maintained at 37°C with 5% CO_2_.

### *In silico* analyses for selection of protospacers.

For knockout and tagging of *TcMPC* genes, we used the genome editing method by CRISPR/Cas9 for T. cruzi developed before (26, 28, 53). Selection of protospacers for both strategies was performed using EuPaGDT (eukaryotic pathogen CRISPR guide RNA/DNA design tool; http://grna.ctegd.uga.edu) (54).

### Endogenous C-terminal tagging of TcMPCs.

CRISPR/Cas9-mediated endogenous C-terminal tagging of TcMPC proteins was performed as previously described (26, 27). To perform individual tagging of *TcMPC1* and *TcMPC2*, we chose protospacer targeting regions close to the 3′ end of the putative *TcMPC1* gene (294 bp, TriTrypDB gene ID, TcCLB.511577.144) and the putative *TcMPC2* gene (414 bp, TriTrypDB gene ID, TcCLB.508265.44). sgRNAs were amplified by PCR using a forward oligonucleotide (primers 1 and 2, Table S1 in the supplemental material) and a common reverse primer (Rv_sgRNA, primer 3, Table S1) and further cloned into the Cas9/pTREX-n vector. To amplify DNA donor cassettes containing 3×c-Myc tag sequence and marker for puromycin resistance (*Pac* gene), a set of long primers (ultramers) (primers 5 to 8, Table S1) for each gene was designed. DNA donors were amplified by PCR using pMOTag23M vector as the DNA template. Wild-type T. cruzi epimastigotes (Y strain) were cotransfected with the gene-specific sgRNA/Cas9/pTREX-n plasmid and linear DNA donor. After 6 weeks of selection with G418 and puromycin, tagging of *TcMPC1* and *TcMPC2* was confirmed by PCR analysis using specific primer sets (primers 9 to 16, Table S1) and by Western blotting. A scrambled sgRNA cell line was used as control (53).

### Knockout of *TcMPCs*.

Molecular construction of sgRNA/Cas9/pTREX-n vector was performed as previously described (28). For knocking out *TcMPC1* and *TcMPC2* individually, we chose protospacer sequences closest to the 5′ end of the gene of interest (GOI), and sgRNAs were amplified by PCR using a forward oligonucleotide (primers 17 and 18, Table S1) and a common reverse primer (Rv_sgRNA, primer 3, Table S1). Ultramers (primers 19 to 22, Table S1) for each gene were designed to amplify DNA donors containing the blasticidin resistance gene (*Bsd*) using pGEM-*Bsd* vector as the DNA template. Wild-type T. cruzi epimastigotes (Y strain) were cotransfected with *TcMPC*-specific sgRNA/Cas9/pTREX-n vector and linear DNA donor. A scrambled sgRNA cell line was used as the control. After 6 weeks of selection with G418 and blasticidin, knockout of *TcMPC1* and *TcMPC2* was confirmed by PCR analysis using specific primer sets (primers 23 to 32, Table S1) and by Southern blotting.

### Overexpression of TcMPC proteins.

For overexpression of 3×HA-tagged TcMPC1 and TcMPC2 proteins, the primers 33 to 36 (Table S1) were designed (XbaI and XhoI restriction sites in the primers forward and reverse, respectively). The pTREX-n/3×HA vector, which provides resistance to G418, and PCR products were doubly digested with XbaI and XhoI. The inserts were independently cloned into the digested pTREX-n/3×HA vector using T4 DNA Ligase, generating pTREX-n/*TcMPC1-*3×HA and pTREX-n/*TcMPC2-*3×HA plasmids. The recombinant constructs were confirmed by PCR analysis with Fw_HX1-pTREX primer (primer 37, Table S1) and the gene-specific reverse primer, digestion with the restriction enzymes, and sequencing. Wild-type T. cruzi epimastigotes (Y strain) were transfected with recombinant constructs. Epimastigotes transfected with pTREX-n/3×HA empty vector were used as the control. After 6 weeks of selection with G418, overexpression of TcMPC1 or TcMPC2 was confirmed by Western blot analysis.

### Cell transfections.

Y strain wild-type T. cruzi epimastigotes were used for cotransfection of the sgRNA/Cas9/pTREX-n plasmid and linear DNA donor. Cell transfections were performed as reported previously (53) in triplicates. Negative controls were flasks containing cells without plasmid DNA that were subjected to the electroporation, and flasks containing cells without plasmid DNA that were not subjected to electroporation.

Transfectants *TcMPC1*-TAG and *TcMPC2*-TAG were cultured in LIT medium supplemented with 20% heat-inactivated FBS, penicillin, and streptomycin plus 250 μg ml^−1^ G418 and 5 μg ml^−1^ puromycin until stable cell lines were obtained. Transfectants *TcMPC1*-KO and *TcMPC2*-KO were cultured in the same medium plus 250 μg ml^−1^ G418 and 10 μg ml^−1^ blasticidin until stable cell lines were obtained.

For transfection with the overexpression vectors pTREX-n/*TcMPC1-*3×HA and pTREX-n/*TcMPC2-*3×HA, we followed the protocol described above using 25 μg of the plasmid. Transfectants *TcMPC1*-OE and *TcMPC2*-OE were cultured in LIT medium supplemented with 20% heat-inactivated FBS, penicillin, and streptomycin plus 250 μg ml^−1^ G418.

### Southern blot analyses.

To corroborate *TcMPC1* and *TcMPC2* gene knockouts by Southern blotting, 25 μg of gDNA from scrambled sgRNA and TcMPC1-KO cell lines was digested with HindIII enzyme and 25 μg of gDNA from scrambled sgRNA and *TcMPC2*-KO cell lines was digested with BamHI enzyme. Products of restriction enzyme digestion were resolved on a 1% agarose gel that was further subjected to alkaline blotting using a nylon membrane (Zeta-Probe GT membrane) according to the manufacturer’s recommendations. The next steps were performed with a North2South chemiluminescent hybridization and detection kit. Probes for hybridization were amplified by PCR using the same primer sets used for the overexpression strategy (primers 33 to 36, Table S1) and pTREX-n/*TcMPC1-*3×HA or pTREX-n/*TcMPC2-*3×HA vector as the DNA template. DNA probes were biotinylated using North2South biotin random prime labeling kit.

### Western blot analyses.

To confirm the expression of the proteins TcMPC1-3×c-Myc, TcMPC2-3×c-Myc, TcMPC1-3×HA, and TcMPC2-3×HA, mid-log-phase epimastigotes from the respective cell lines *TcMPC1*-TAG, *TcMPC2*-TAG, *TcMPC1*-OE, and *TcMPC2*-OE were harvested by centrifugation at 1,000 × *g* for 7 min and washed twice with phosphate-buffered saline (PBS), pH 7.4, at room temperature (RT). The pellets were resuspended in 100 μl of RIPA buffer (150 mM NaCl, 20 mM Tris-HCl, pH 7.5, 1 mM EDTA, 1% sodium dodecyl sulfate [SDS], and 0.1% Triton X-100) plus 1 mM PMSF, 2.5 mM TPCK, 100 μM E64, and protease inhibitor cocktail (P8340, 1:250 dilution). Samples were incubated on ice for 1 h and then homogenized with a 1-ml syringe. Lysates were kept at −80°C until further use. Protein concentration in the lysates was determined using the BCA protein assay kit. Approximately 50 μg of each lysate was mixed with 6× Laemmli sample buffer (300 mM Tris-HCl, pH 6.8, 6% [wt/vol] β-mercaptoethanol, 50% [vol/vol] glycerol, 12% [wt/vol] SDS, 4% [wt/vol] bromophenol blue) and then subjected to electrophoresis using a 12% SDS-polyacrylamide gel electrophoresis (SDS-PAGE). Precision Plus protein dual color standard was applied on the gel. Electrophoresed proteins were transferred to a nitrocellulose membrane using a Bio-Rad transblot apparatus for 1 h at 100 V at 4°C. Following transfer, the membrane blots were blocked with 5% nonfat dry milk in PBS containing 0.1% (vol/vol) Tween 20 (PBS-T) at 4°C overnight. Blots were probed with mouse anti-c-Myc monoclonal antibody (1:100 dilution in PBS-T), mouse anti-HA monoclonal antibody (1:5,000 dilution in PBS-T), or mouse anti-tubulin monoclonal antibody (1:40,000 dilution in PBS-T) for 1 h at RT. After washing three times with PBS-T, the blots were incubated with horseradish peroxidase (HRP)-conjugated goat anti-mouse IgG antibody (1:10,000 dilution in PBS-T) for 1 h at RT. The membranes were washed three times with PBS-T, and Western blot images were processed and analyzed using SuperSignal West Pico chemiluminescent substrate and a UVItec Cambridge Alliance 2.7 imaging system (UVItec, Cambridge, United Kingdom) according to the manufacturer’s instructions.

For Western blot analysis to detect phosphorylation of PDH, 30 μg of each lysate, the rabbit polyclonal anti-PDH-E1α (phospho-Ser-293) primary antibody (1:1,000 dilution in PBS-T), and the goat HRP-conjugated anti-rabbit IgG secondary antibody (1:10,000 dilution in PBS-T) were used.

### Immunofluorescence analyses.

For immunofluorescence assays of *TcMPC1-*TAG, *TcMPC2*-TAG, *TcMPC1-*OE, and *TcMPC2*-OE epimastigotes, cells were incubated with 100 nM MitoTracker Deep Red FM in culture medium for 30 min at 28°C. After staining with MitoTracker dye, cells were washed twice with PBS, pH 7.4 (PBS1), and fixed with 4% paraformaldehyde in PBS1 for 1 h at RT. Then, cells were allowed to adhere to poly-l-lysine-coated coverslips for 30 min. The coverslips were washed three times with PBS1 and incubated with 0.3% Triton X-100 in PBS1 for 3 min. Permeabilized cells were washed twice with PBS1 and blocked with PBS1 containing 5% goat serum, 50 mM NH_4_Cl, 3% bovine serum albumin (BSA), and 1% fish gelatin at 4°C overnight. After blocking, cells were incubated with a primary antibody, mouse anti-c-Myc monoclonal antibody (1:10 dilution, 1% BSA in PBS, pH 8.0 [PBS2]) for *TcMPC*-TAG epimastigotes or mouse anti-HA monoclonal antibody (1:500 dilution in PBS2) for *TcMPC*-OE epimastigotes, for 1 h at RT. The coverslips were washed three times with 1% BSA in PBS2 and then incubated with Alexa Fluor 488-conjugated goat anti-mouse antibody (1:1,000 dilution) diluted in 1% BSA in PBS2 for 1 h at RT in the dark. Cells were washed three times with 1% BSA in PBS2, washed once with PBS2, and counterstained with 4′,6-diamidino-2-phenylindole (DAPI; 5 μg ml^−1^) in Fluoromount-G mounting medium on the slides. Negative controls were performed as described above but in the absence of the primary antibody. Differential interference contrast and fluorescence optical images were captured with a 100× oil immersion lens objective (1.44 aperture) under nonsaturating conditions with a Leica TCS SP5 II confocal microscope, which uses photomultiplier tubes for the detection of emission. Digital images were acquired and processed with Leica LAS AF Lite software (Leica, Wetzlar, Germany).

### Metacyclogenesis assay.

The protocol described by Bourguignon et al. (55) was followed with minor modifications (20). Cells were harvested from TAU 3AAG medium at day 5 of cultivation. Three independent experiments were conducted.

### Invasion and replication assays.

Gamma-irradiated (2,000 radiation-absorbed doses) Vero cells (4.5 × 10^5^ cells) were plated onto sterile coverslips in a 12-well plate and incubated overnight at 35°C with 7% CO_2_ in RPMI medium supplemented with 10% fresh FBS. Tissue culture-derived trypomastigote collections were incubated at 4°C overnight to allow amastigotes to settle from swimming trypomastigotes. Trypomastigotes from the supernatants of these collections were counted and used to infect the mammalian cells at a 10:1 ratio of parasites to host cells. At 4 h postinfection, coverslips were washed extensively with Hank’s balanced salt solution, followed by PBS, pH 7.4, to remove any extracellular parasites. This protocol has been optimized with a multiplicity of infection (MOI) of 10 to ensure that, at most, one trypomastigote (Y strain) invades each irradiated host cell. Coverslips were immediately fixed in 4% paraformaldehyde in PBS, pH 7.4, at 4°C for 30 min and then washed once with PBS and counterstained with DAPI (15 μg ml^−1^) in Fluoromount-G mounting medium on glass slides, which stains host and parasite DNA. Coverslips were analyzed on an Olympus BX60 microscope to quantify the number of host cells that contained intracellular parasites and the number of intracellular parasites per cell in 40 randomly selected fields. Four hundred host cells were counted per sample in three independent experiments. To quantify amastigote replication, the following modifications were used: Vero cells were infected at a 10:1 ratio of parasites to host cells, and after washing them at 4 h postinfection as described above, coverslips were allowed to incubate for 48 h postinfection at 35°C with 7% CO_2_ prior to fixation and DAPI staining.

### Oxygen consumption assays.

The oxygen consumption rate (OCR) of digitonin-permeabilized epimastigotes was measured using a high-resolution Oroboros Oxygraph-2k oxygraph (Oroboros Instruments GmbH, Innsbruck, Austria) with DatLab 4 software for data acquisition and analysis. The equipment was calibrated as reported by its manufacturer. Parasites from the cultures for wild-type, scrambled sgRNA, *TcMPC1*-KO, and *TcMPC2*-KO (1 × 10^8^ cells), pTREX-n/3×HA, *TcMPC1*-OE, and *TcMPC2*-OE (8.5 × 10^7^ cells) were washed with buffer A with glucose (BAG; 116 mM NaCl, 5.4 mM KCl, 0.8 mM MgSO_4_, 50 mM HEPES, pH 7.2, and 5.5 mM glucose). Then, cells were incubated at 28°C and 750 rpm in a 2-ml chamber containing 125 mM sucrose, 65 mM KCl, 10 mM HEPES-KOH, pH 7.2, 1 mM MgCl_2,_ 2.5 mM K_2_HPO_4_, 0.2% BSA, 50 μM EGTA, and 5 mM mitochondrial substrate (succinate, pyruvate, or α-ketoglutarate, as indicated in the figure legends). Sequential additions of digitonin (25 μM), ADP (100 μM), oligomycin (1 μg ml^−1^), and FCCP (1 μM) were performed during reading time. Inhibitors UK5099 (50 μM) and malonate (2 mM) were independently added into the chamber prior to reading in each experiment of inhibition using pyruvate as the mitochondrial substrate. OCR was calculated as the negative time derivative of O_2_ concentration measured in the closed oxygraph chambers and expressed per milligram of protein. Data were recorded at 2-s intervals, and 10 data points were used to calculate the OCR slope plot through a polynomial fit with DatLab 4 software, as previously described (56). For comparison between OCRs of mutant cells and OCRs of control cells, normalized OCR was calculated from the normalization of OCR after an addition of ADP, oligomycin, or FCCP in relation to the basal OCR obtained right before ADP addition. A normalized OCR of 1 means that OCR after an addition is equal to the basal OCR; *n* independent biological experiments are indicated in the respective figure legends.

### Mitochondrial calcium uptake assay.

Mitochondrial Ca^2+^ uptake in permeabilized scrambled, *TcMPC1*-KO, and *TcMPC2*-KO epimastigotes was assayed by fluorescence of calcium green-5N probe at 28°C, as described previously (53), with the addition of 5 mM succinate or pyruvate to the reaction buffer (as indicated in the figures). Three independent experiments were performed.

### Mitochondrial membrane potential assay.

Mitochondrial membrane potential was assessed spectrofluorometrically using the indicator dye safranine O, as described previously (53, 57) with the following modification: the reaction buffer contained 5 mM pyruvate or succinate (as indicated in the figure legends). Additions of ADP (250 μM), CAT (1.5 μM), and FCCP (4 μM) were performed at different time points. The adenine nucleotide translocator inhibitor CAT was used in the membrane potential assay instead of oligomycin, because the basal fluorescence is only reached with CAT following the addition of ADP. Three independent experiments were performed.

### α-Ketoglutarate dehydrogenase activity assay.

The measurements of α-ketoglutarate dehydrogenase (α-KGDH) activity in control, *TcMPC1*-KO, and *TcMPC2*-KO epimastigotes were performed as described previously (58) with some modifications. Briefly, protein extracts from scrambled, *TcMPC1*-KO, and *TcMPC2*-KO epimastigotes were obtained by centrifugation of 10 ml of culture in exponential phase (1 × 10^7^ to 2 × 10^7^ cells ml^−1^), washed with PBS, pH 7.4, at RT, and resuspended in 100 μl lysis buffer (0.1% Triton X-100, 50 mM K_2_HPO_4_ [pH 7.35] and 5 U ml^−1^ Benzonase). Protein extracts were incubated for 10 min on ice before protein quantification with a BCA protein assay kit. The conversion of α-ketoglutarate, NAD^+^, and coenzyme A to succinyl-CoA, NADH, and CO_2_ catalyzed by α-KGDH was measured in 96-well plates by following NADH fluorescence for 10 min at wavelengths of 340 and 460 nm for excitation and emission, respectively, using a Synergy H1 hybrid reader (BioTek, Winooski, VT). The α-KGDH activity assay was carried out in reaction buffer containing 50 mM K_2_HPO_4_ (pH 7.35), 1 mM MgCl_2_, 0.2 mM thiamine pyrophosphate, 0.3 mM dithiothreitol, 100 μM EGTA, 50 μM coenzyme A-SH, 2 mM NAD^+^, 1 mM α-ketoglutarate, and 5 μg protein extract in a final volume of 260 μl, at RT. Blank wells containing lysis buffer instead of protein extracts were included in each assay, and their mean fluorescence was subtracted from that in the reaction wells. *V*_max_ values were used to normalize the α-KGDH activity from each cell line using control parasites as a reference sample. Three independent experiments were performed.

### Statistical analyses.

Values are expressed as means ± standard errors of the means (SEMs). Significant differences between treatments were compared using one-way and two-way analysis of variance (ANOVA) tests and the Kruskal-Wallis test, where indicated. Differences were considered statistically significant at *P* value of <0.05, and *n* refers to the number of experiments performed. All statistical analyses were conducted using GraphPad Prism 6 (GraphPad Software, San Diego, CA).
