# Supplementary figures and images for: Mitochondrial Pyruvate Carrier Subunits Are Essential for Pyruvate-Driven Respiration, Infectivity, and Intracellular Replication of Trypanosoma cruzi
Source: mBio. 2021 Apr 6;12(2):e00540-21. doi: 10.1128/mBio.00540-21 (PMC8092248; doi:10.1128/mBio.00540-21)

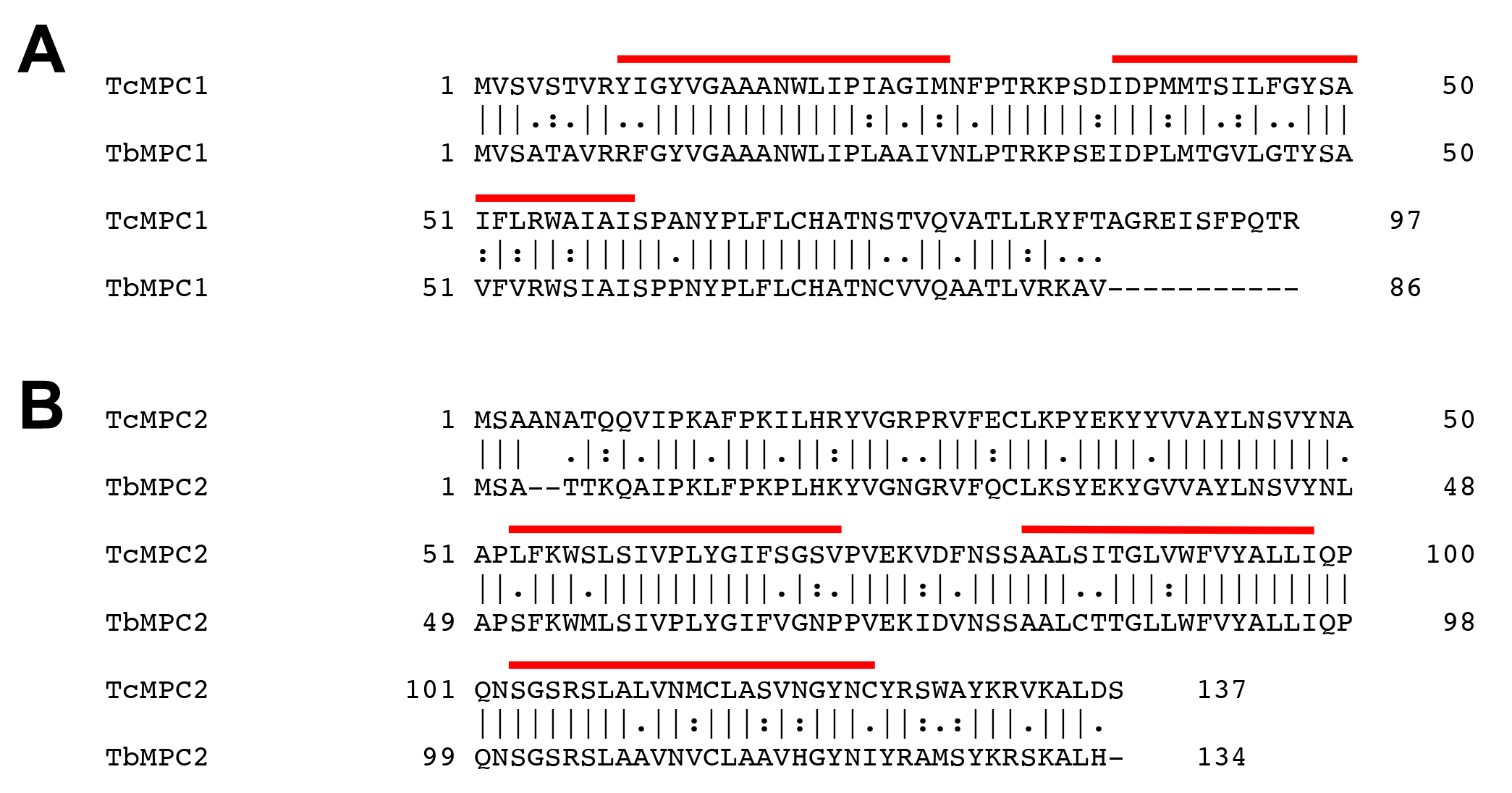

Supplement: FIG S1 [file mBio.00540-21-sf001.tif]

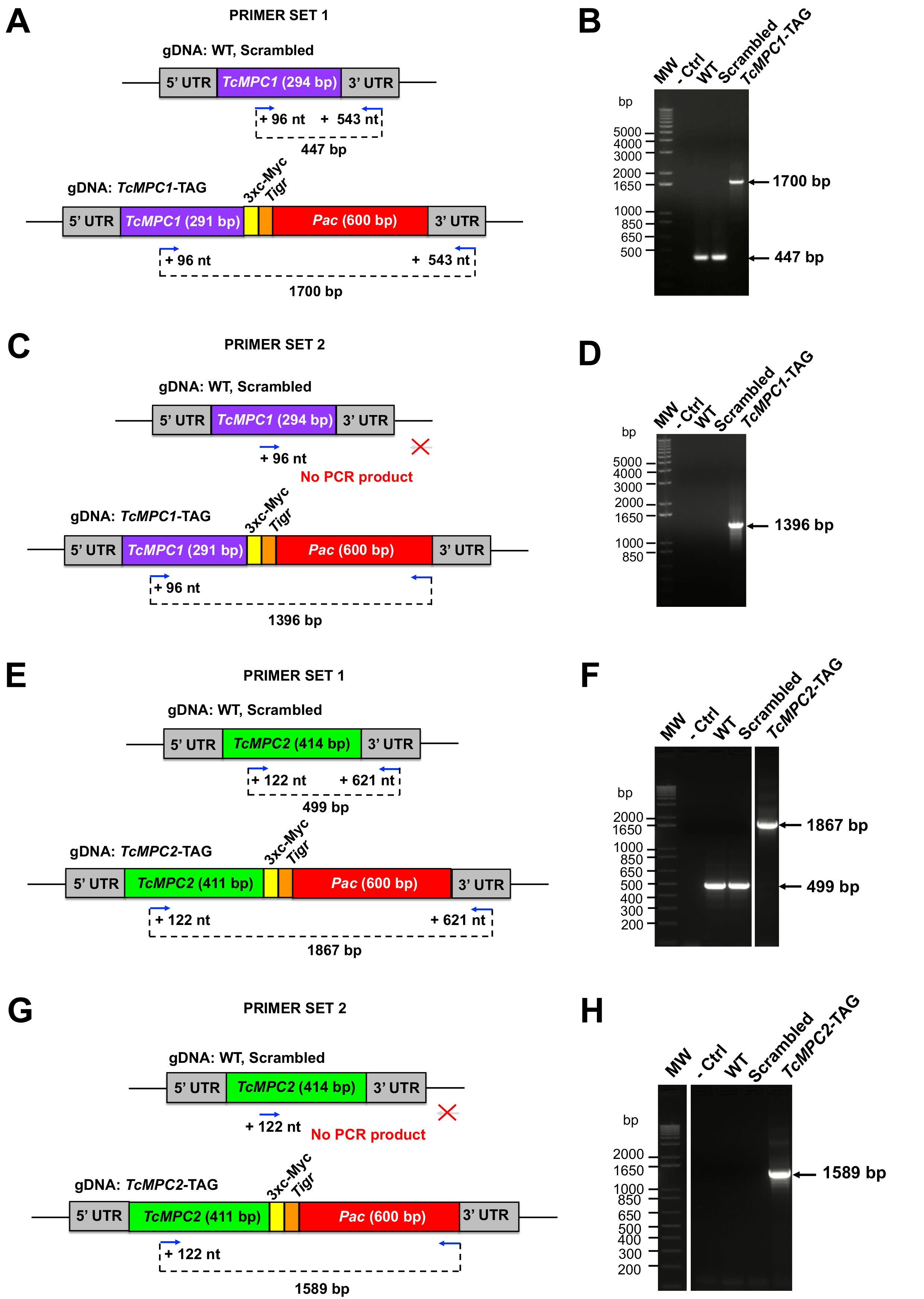

Supplement: FIG S2 [file mBio.00540-21-sf002.tif]

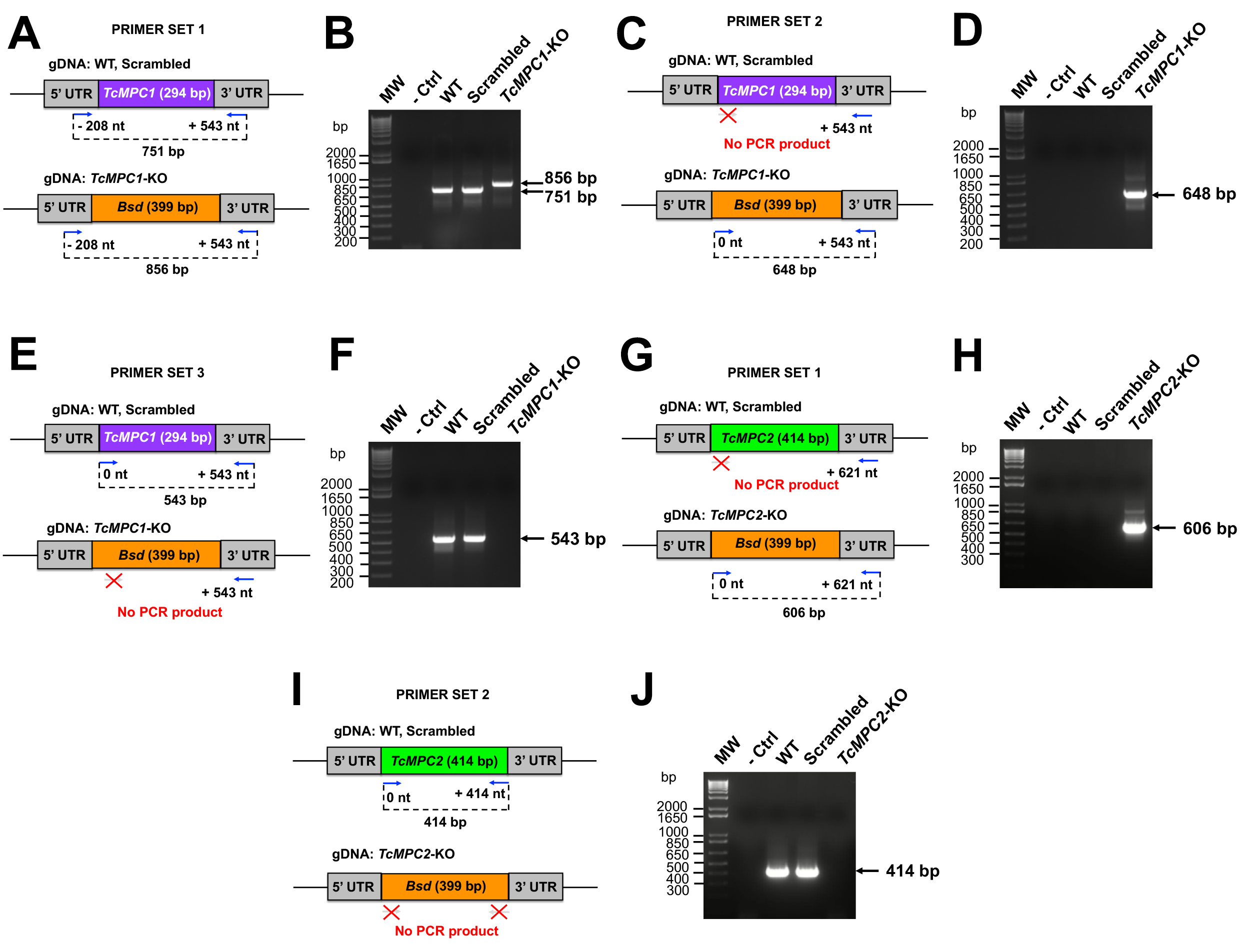

Supplement: FIG S3 [file mBio.00540-21-sf003.tif]

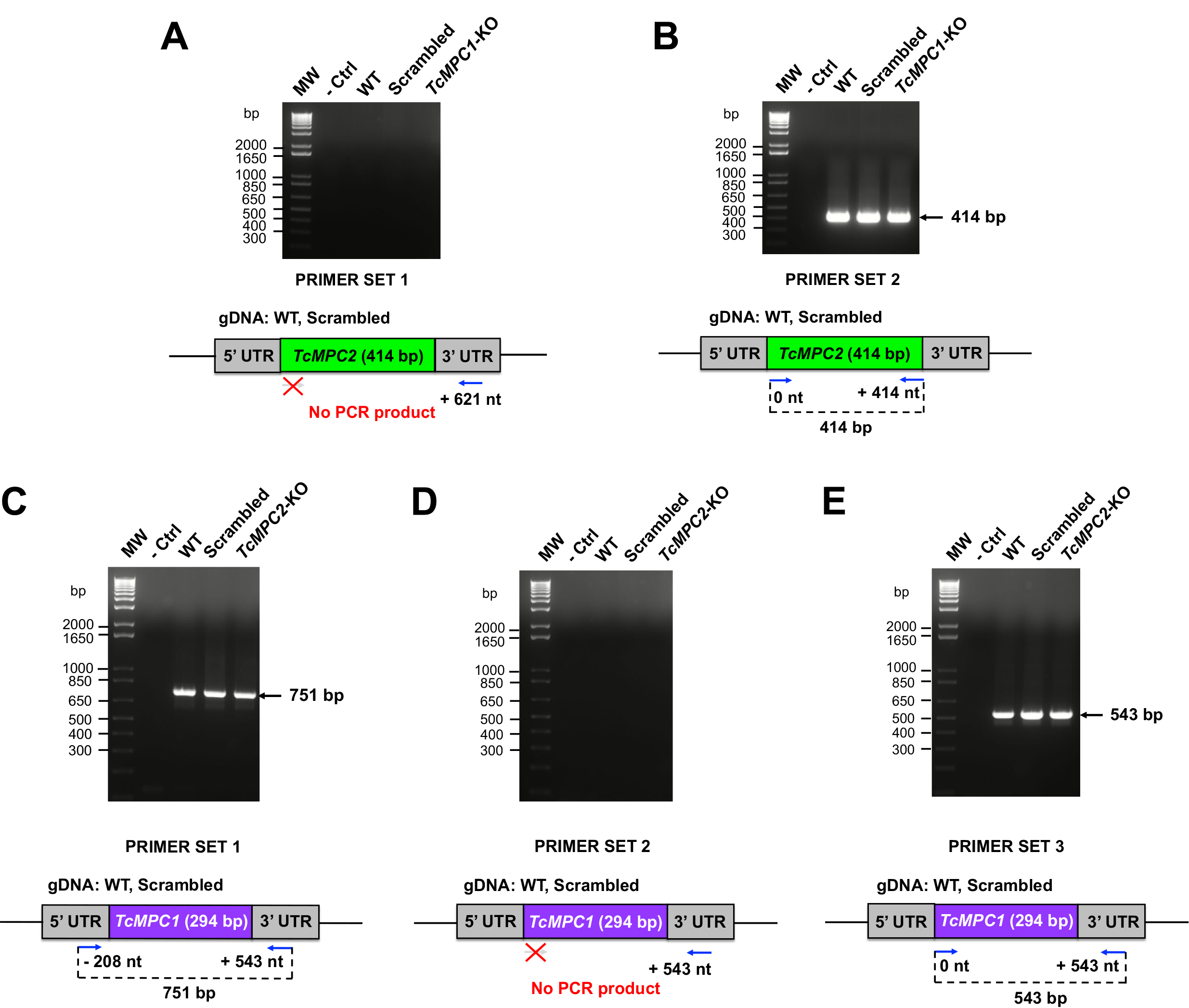

Supplement: FIG S4 [file mBio.00540-21-sf004.tif]

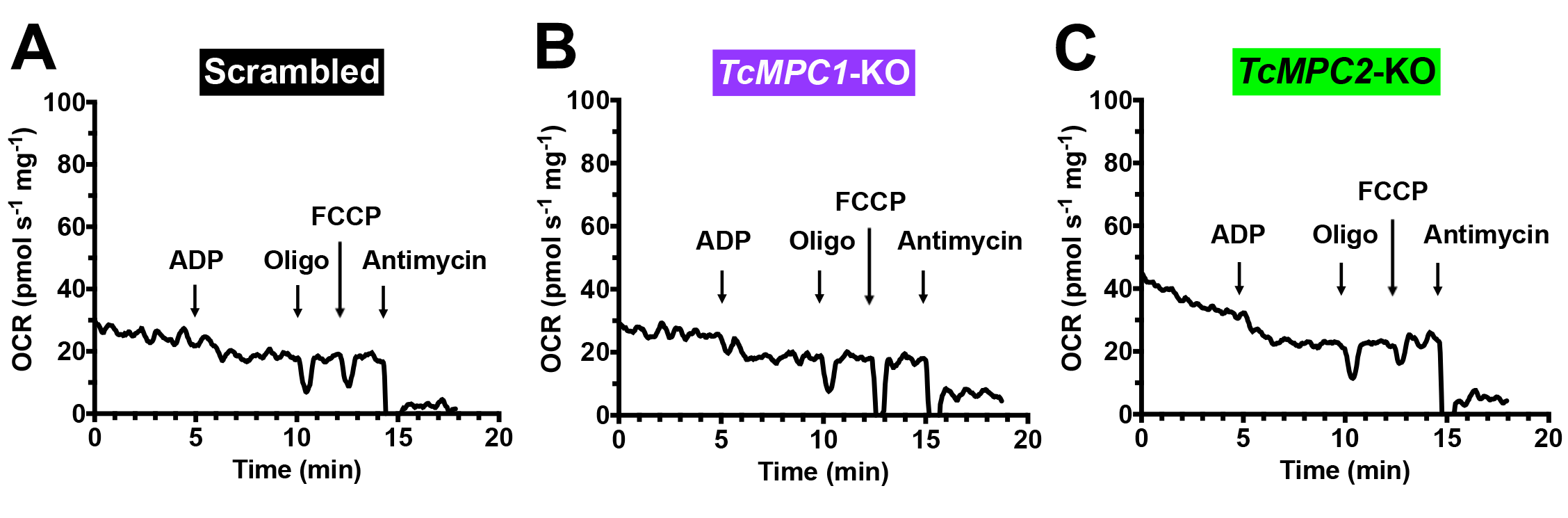

Supplement: FIG S5 [file mBio.00540-21-sf005.tif]

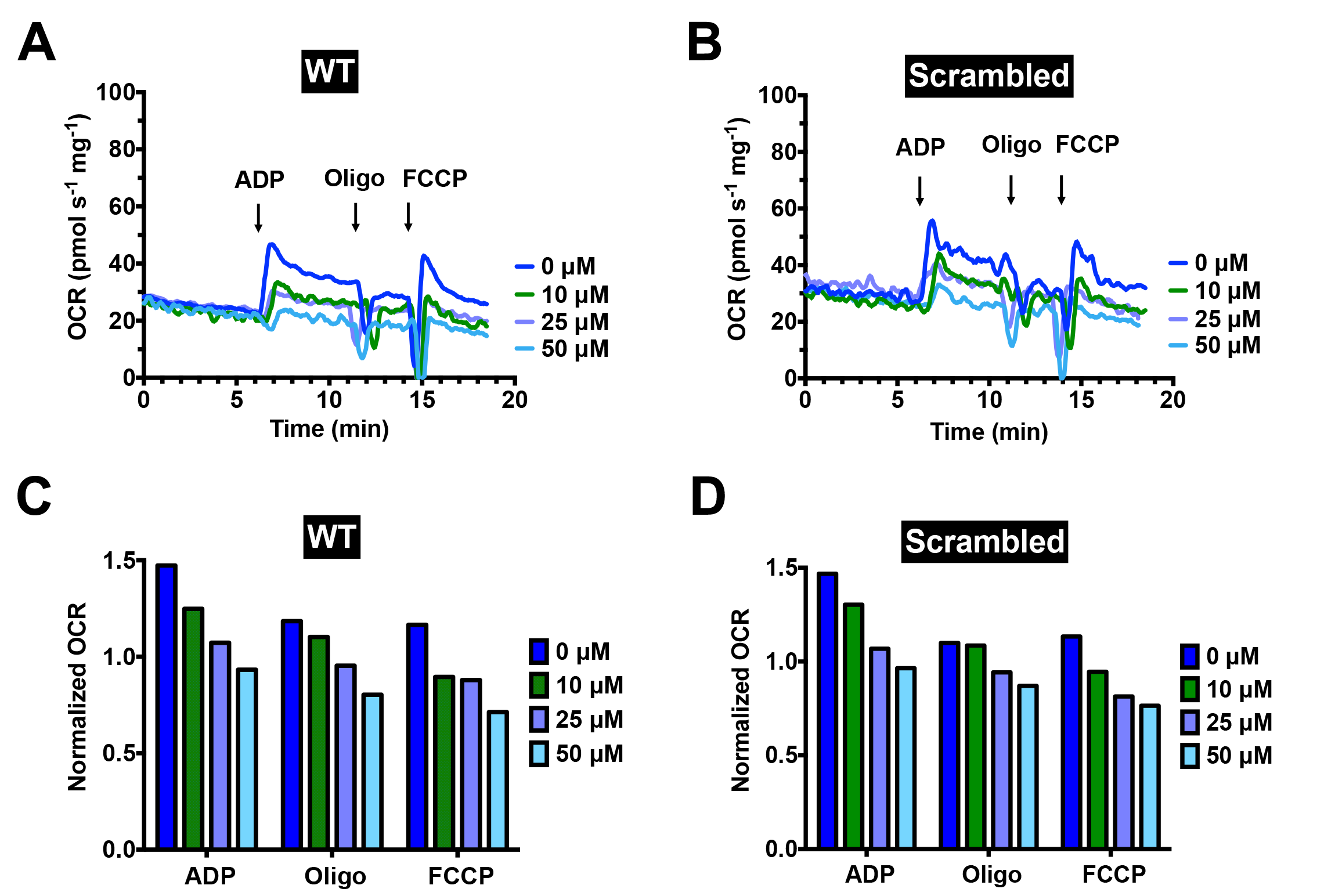

Supplement: FIG S6 [file mBio.00540-21-sf006.tif]

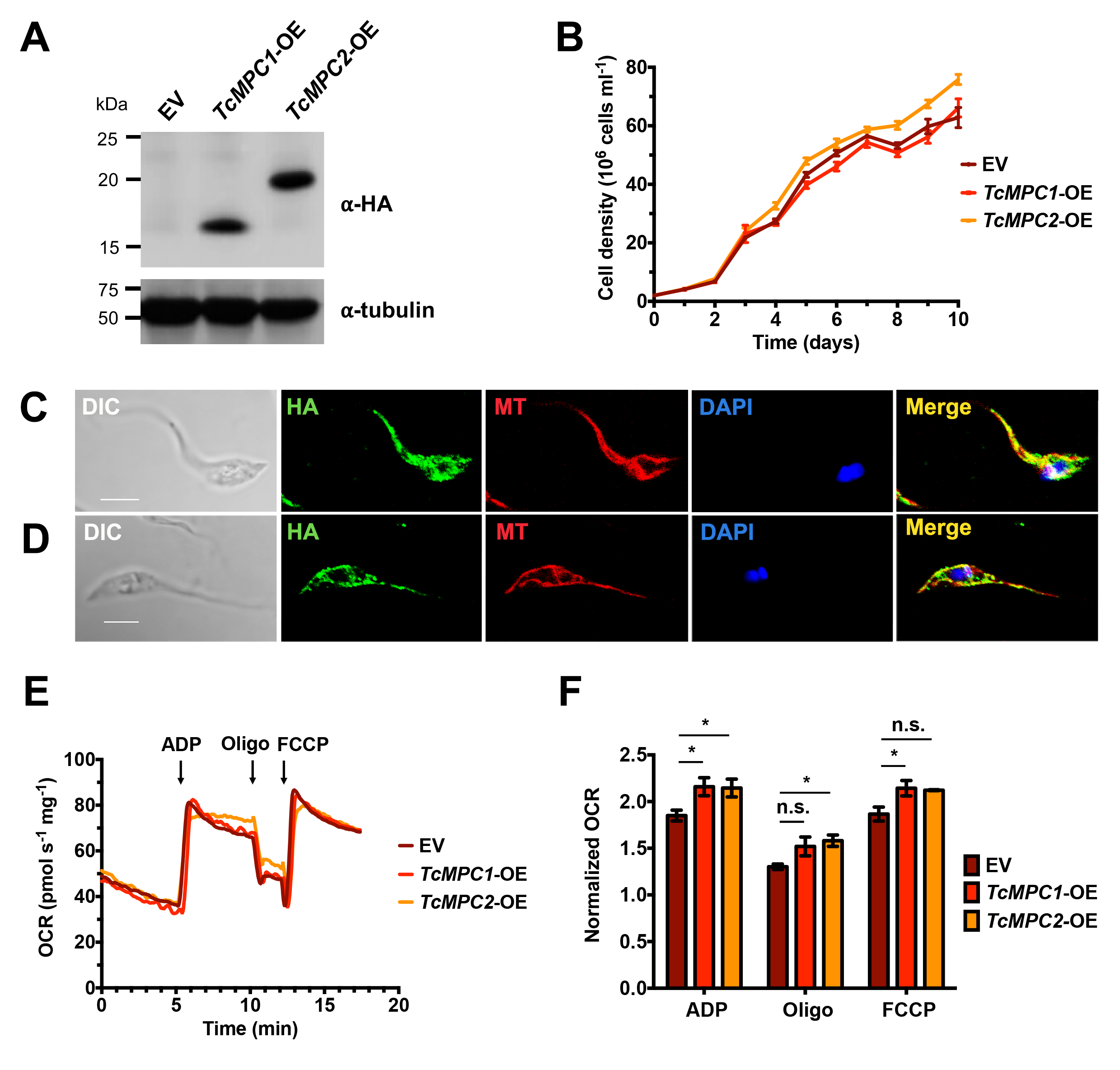

Supplement: FIG S7 [file mBio.00540-21-sf007.tif]
