# Supplementary material for: Mitochondrial Pyruvate Carrier Subunits Are Essential for Pyruvate-Driven Respiration, Infectivity, and Intracellular Replication of Trypanosoma cruzi
Source: mBio. 2021 Apr 6;12(2):e00540-21. doi: 10.1128/mBio.00540-21 (PMC8092248; doi:10.1128/mBio.00540-21)
Supplement: TABLE S1 [file mBio.00540-21-st001.docx]

**Table S1.** List of oligonucleotides used in this study.

| No. | Primer name | Nucleotide sequence (5' → 3') |  |
| --- | --- | --- | --- |
| 1 | Fw_TcMPC1-TAG | GATC**GGATCC**ACACAATCAGACTTTTTTAGGTTTTAGAGCTAGAAATAGC | |
| 2 | Fw_TcMPC2-TAG | GATC**GGATCC**ACAGTTAGCAACCACAATCGGTTTTAGAGCTAGAAATAGC | |
| 3 | Rv_sgRNA | CAGT**GGATCC**AAAAAAGCACCGACTCGGTG | |
| 4 | Rv_HX1-pTREX | TAATTTCGCTTTCGTGCGTG | |
| 5 | Fw_TcMPC1-TAG_ultramer | CCCCTTATTTTTGTGTCATGCCACGAATAGTACGGTGCAGGTTGCAACTCTTTTGCGCTATTTTACAGCTGGAAGAGAGATTTCTTTTCCCCAGACGCGGGGTACCGGGCCCCCCCTCGAG | |
| 6 | Rv_TcMPC1-TAG_ultramer | CTGGTGGCACTCAAAAACAGAAACAGTAAAAGTAAGCCCAAATGGCAGTTAACACAAAAAAAAAGGAAACGATATGTCCTTTTTTTTTCCCCCTAACCCTTGGCGGCCGCTCTAGAACTAGTGGAT | |
| 7 | Fw_TcMPC2-TAG_ultramer | GAGTCGTTCATTGGCTCTCGTTAACATGTGTCTGGCTTCCGTAAACGGGTACAACTGCTACCGCAGCTGGGCCTACAAGAGAGTCAAGGCATTGGACAGTGGTACCGGGCCCCCCCTCGAG | |
| 8 | Rv_TcMPC2-TAG_ultramer | TATATAAATGTATTAATACATGTGTATGTAACCCATCTCCTCATCCTCTTTTTATTCTCCTTTGGGTTTATGTGACGGAGTGCGGTTGCAAGTTTGACCTTGGCGGCCGCTCTAGAACTAGTGGAT | |
| 9 | Fw_TcMPC1-TAG_set1_check | GAAGCCCAGTGACATTGATC | |
| 10 | Rv_ TcMPC1-TAG_set1_check | GCAAAGAAAAACCTTTGCGTAC | |
| 11 | Fw_TcMPC1-TAG_set2_check | GAAGCCCAGTGACATTGATC | |
| 12 | Rv_ TcMPC1-TAG_set2_check | TCAGGCACCGGGCTTGCGGG | |
| 13 | Fw_TcMPC2-TAG_set1_check | GGCCTACTTGAACTCCGTTTAC | |
| 14 | Rv_ TcMPC2-TAG_set1_check | CACCATGTAACACGAAGTAAG | |
| 15 | Fw_TcMPC2-TAG_set2_check | GGCCTACTTGAACTCCGTTTAC | |
| 16 | Rv_ TcMPC2-TAG_set2_check | TCAGGCACCGGGCTTGCGGG | |
| 17 | Fw_TcMPC1-KO | GATC**GGATCC**GTAAGCACTGTCCGTTACATGTTTTAGAGCTAGAAATAGC | |
| 18 | Fw_TcMPC2-KO | GATC**GGATCC**TGCTGCCAACGCTACACAGCGTTTTAGAGCTAGAAATAGC | |
| 19 | Fw_TcMPC1-KO_ultramer | TCGTCCTTTCATCTTAATTACTTTTTCTATTTATTTATTTATTTACTTTTTTGTTTGAAC  TAGATCAAAAGGAAAGGTACTTATAAGTATATATTATTCAATGGCCAAGCCTTTGTCTCA | |
| 20 | Rv_TcMPC1-KO_ultramer | AAAAGTAAGCCCAAATGGCAGTTAACACAAAAAAAAAGGAAACGATATGTCCTTTTTTTTTCCCCCTAACCCTCTAAAAAAGTCTGATTGTGTGCAATAATTAGCCCTCCCACACATAAC | |
| 21 | Fw_TcMPC2-KO_ultramer | TTTCCTTTTTCTTTTTGGACTTACTAGTTAACTTGACGAAGAAGAAAAAAAAAAAAAAAA  AAAAAACAGTGGGGAAGGGAAAAGTTTGGGGAAAAGAAACATGGCCAAGCCTTTGTCTCA | |
| 22 | Rv_TcMPC2-KO_ultramer | TTAATACATGTGTATGTAACCCATCTCCTCATCCTCTTTTTATTCTCCTTTGGGTTTATGTGACGGAGTGCGGTTGCAAGTTTGACCTCGATTGTGGTTGTTAGCCCTCCCACACATAAC | |
| 23 | Fw_TcMPC1-KO_set1_check | GATGTAGAGAAGAATTTGTTGAGG | |
| 24 | Rv_ TcMPC1-KO_set1_check | GCAAAGAAAAACCTTTGCGTAC | |
| 25 | Fw_TcMPC1-KO_set2_check | ATGGCCAAGCCTTTGTCTCAAG | |
| 26 | Rv_ TcMPC1-KO_set2_check | GCAAAGAAAAACCTTTGCGTAC | |
| 27 | Fw_TcMPC1-KO_set3_check | ATGGTTTCCGTAAGCACTGTCCG | |
| 28 | Rv_ TcMPC1-KO_set3_check | GCAAAGAAAAACCTTTGCGTAC | |
| 29 | Fw_TcMPC2-KO_set1_check | ATGGCCAAGCCTTTGTCTCAAG | |
| 30 | Rv_ TcMPC2-KO_set1_check | CACCATGTAACACGAAGTAAG | |
| 31 | Fw_TcMPC2-KO_set2_check | ATGTCTGCTGCCAACGCTACAC | |
| 32 | Rv_ TcMPC2-KO_set2_check | CTAACTGTCCAATGCCTTGAC | |
| 33 | Fw_TcMPC1-OE | GC**TCTAGA**ATGGTTTCCGTAAGCACTGTCCG | |
| 34 | Rv_TcMPC1-OE | CCG**CTCGAG**CCGCGTCTGGGGAAAAGAAATC | |
| 35 | Fw_TcMPC2-OE | GC**TCTAGA**ATGTCTGCTGCCAACGCTACAC | |
| 36 | Rv_TcMPC2-OE | CCG**CTCGAG**ACTGTCCAATGCCTTGACTCTCTTG | |
| 37 | Fw_HX1-pTREX | CATTTTCACGCACGAAAGC | |

Restriction sites are indicated in bold. Protospacers are underlined.

Primers: 1 to 16 for tagging, 17 to 32 for knockout, and 33 to 37 for overexpression strategy.
